# Molecular genetic analysis of two native desert palm genera, *Washingtonia* and *Brahea*, from the Baja California Peninsula and Guadalupe Island

**DOI:** 10.1002/ece3.3036

**Published:** 2017-05-30

**Authors:** Anastasia Klimova, Joseph I. Hoffman, Jesus N. Gutierrez‐Rivera, Jose Leon de la Luz, Alfredo Ortega‐Rubio

**Affiliations:** ^1^ Centro de Investigaciones Biologicas del Noroeste S.C.La PazBaja California SurMexico; ^2^ Department of Animal BehaviourUniversity of BielefeldBielefeldGermany

**Keywords:** Baja California Peninsula, desert fan palms, relict species, species distributions

## Abstract

The complex geological and ecological processes that have generated high levels of biodiversity and endemism in the Baja California Peninsula have been the subject of intensive study. However, relatively little is known about phylogeography of the iconic endemic palm species of this region. We therefore analyzed a total of 2,294 bp of chloroplast and 738 bp of nuclear sequence data in 169 samples of five native palm species from Baja California, Sonora and Guadalupe Island. We found that *Washingtonia* and *Brahea* palms had low levels of genetic diversity and were highly structured, with the majority of species and major geographic regions being characterized by distinct haplotypes. We also found strong support for currently recognized species in *Washingtonia*, but our results were less clear cut for *Brahea* due to haplotype sharing. Furthermore, patterns of population structure were broadly consistent with historical vicariant events such as the inundation of the Isthmus of La Paz, the formation of the Sea of Cortez, and the more recent colonization and isolation of Guadalupe Island's palms. Our findings contribute toward a growing appreciation of the complexity of plant responses to past geological changes and also provide valuable baseline genetic data on relict American palm species.

## 1 INTRODUCTION

Spatial patterns of species abundance and distribution are of a fundamental importance as they contribute toward virtually every facet of ecological knowledge from conservation to the understanding of ecosystem function and structure. Current species distributions arise from the interplay of various evolutionary, ecological and geological forces as well as from stochastic effects acting on species and populations. Multiple factors have been implicated in shaping species distributions including contemporary climate conditions (Currie et al., 2004; Svenning & Skov, 2007), Quaternary climatic oscillations (Hewitt, 1996, 2000, 2004), topography and habitat heterogeneity (Kerr & Packer, 1997), dispersal capacity (Svenning, Normand, & Skov, 2008), and biotic interactions (Araújo & Luoto, 2007; Kissling, Rahbek, & Bohning‐Gaese, 2007).

The biological diversity of the Baja California Peninsula has long been recognized as unique and significant (Grismer, 2000; Lindell, Ngo, & Murphy, 2006; Riemann & Ezcurra, 2005; Wiggins, 1980). The Baja California is also considered a “natural laboratory” in which multiple geological and ecological events are thought to have triggered cycles of vicariant episodes followed by dispersion along climatic gradients (Mantooth, Hafner, Bryson, & Riddle, 2013; Riddle, Hafner, Alexander, & Jaeger, 2000). The major geological processes that have been evoked in explaining patterns of biotic diversification in the region have been classified on the basis of their timing and strength (Dolby, Bennett, Lira‐Noriega, Wilder, & Munguia‐Vega, 2015). The continental rifting of the Peninsula away from mainland Mexico and the volcanic upwelling of Guadalupe Island are the most ancient (>5 million years ago, MYA) and resulted in ecological speciation, island endemism and disjunctions in mainland‐peninsular and peninsular‐island sister species distributions (Aleixandre, Hernandez Montoya, & Mila, 2013; Grismer, 2000; León De La Luz, Rebman, & Oberbauer, 2003; Riddle et al., 2000; Rosas‐Escobar, Gernandt, Pinero, & Garcillan, 2011). More recently (1–3 MYA) and more locally, complex physical interactions between the land and the sea, such as the inundation of the Isthmus of La Paz and the formation of the temporal mid‐peninsular seaway, resulted in a north‐south genetic discontinuity within the peninsula and the generation of regions of high local endemism (Dolby et al., 2015; León‐De la Luz & Breceda, 2006; McCauley, Cortés‐Palomec, & Oyama, 2010; Riddle et al., 2000). This complex interplay was followed by progressive aridification of the Baja Peninsula and adjacent areas after the Last Glacial Maximum (LGM) (Hafner & Riddle, 1997; Lindell et al., 2006; Riddle et al., 2000). Following drastic changes in precipitation, a number of endemic species are thought to have become either locally extinct or restricted to isolated permanent water bodies such as oases and canyons (Grismer & McGuire, 1993; Wehncke, López‐Medellín, & Ezcurra, 2010).

Palms (Arecaceae) are one of the most distinctive of all plant families and are emblematic of the tropics (Tregear, Rival, & Pintaud, 2011). Although the majority of palm species are restricted to the wet tropics, several groups occur in dry open savannas and desert oases (Tomlinson, 2006). Palm populations on the Baja California Peninsula are considered to be relicts of historically more widespread and continuous populations that are now largely confined to sites where permanent water exists either above or below the ground (Axelrod, 1950; Cornett, 1985; Cornett, Glen, & Stewart, 1986; Felger & Joyal, 1999; Grismer & McGuire, 1993). This is evident in the fossil record, which shows that during the late Cretaceous, palms were common across North America and extended much further than their current geographic distribution (Couvreur, Forest, & Baker, 2011; Harley, 2006).

Two native palm genera (*Washingtonia* spp. and *Brahea* spp.) are currently present on the Baja Peninsula (Minnich, Franco‐Vizcaino, & Salazar‐Ceseña, 2011). The genus *Washingtonia* is represented by two species, *W. robusta* and *W. filifera*, but the taxonomical relationships within this genus are poorly resolved (Felger & Broyles, 2007; Felger & Joyal, 1999; Henderson, Galeano, & Bernal, 1995; McClintock, 1978). *W. filifera* is distributed from southeastern California and western Arizona to northern Baja California (McClintock, 1978). By contrast, *W. robusta* is locally distributed near Cataviña and Sierra Asamblea and is more common in the southern half of the Baja peninsula (Minnich et al., 2011). It also occurs on the Mexican mainland, where it is restricted to a few riparian canyons at the southern edge of the Sonoran Desert and appears to be relictual, being geographically and ecologically the most narrowly distributed palm species in the region (Felger & Joyal, 1999). There is still unresolved debate over the relictual status of *Washingtonia* palms, with some authors having suggested that their current distribution is of recent invasive origin (Cornett, 1987b, 2008) or might be linked to the movement of native human populations (Hicks, 1989; Minnich et al., 2011). One the other hand, many characteristics of *Washingtonia* palms such as their patchy geographical distributions, low numbers, and affinity to tropical climates would support fossil evidence in suggesting that their current distribution may be relictual (Axelrod, 1950; Felger & Joyal, 1999).

The *Brahea* complex comprises nine species, two of which are endemic and restricted to Baja California (*B. brandegeei* and *B. armata)* and one (*B. edulis*) to Guadalupe Island. The remaining *Brahea* species are absent from the Baja California Peninsula but can be found on the Mexican mainland, with some species (*B. nitida* and *B. dulcis*) extending their range into Central America. *Brahea* is the least studied genus of American palms and the relationships between and within its species are not clearly described (Henderson et al., 1995). The geographical range of *B. brandegeei* extends from the northern Baja California Sur (Sierra San Francisco) to Sierra La Laguna at the southernmost tip of the peninsula (Felger, Johnson, & Wilson, 2001; Minnich et al., 2011). *B. armata* extends from just south of the United States‐Mexico border southward to just north of the state line of Baja California Sur in the central peninsula (Franco‐Vizcaino, Lopez‐Beltran, & Salazar‐Cesena, 2007; Wiggins, 1980). Nonetheless, the exact limits of the ranges of *B. armata* and *B. brandegeei* are unclear because of taxonomic uncertainties (Felger & Joyal, 1999; Felger et al., 2001; Henderson et al., 1995). The Guadalupe fan palm or *B. edulis* is distributed across the northwestern side of Guadalupe Island (Garcillán, Vega, & Martorell, 2012; León De La Luz et al., 2003) with small patches found along the bottoms of arroyos throughout the island (Oberbauer, 2005).

In spite of the ecological, ethno‐biological and economical importance of *Washingtonia* and *Brahea* palms, many aspects of their biology including species delimitations, genetic relationships between species and levels of intraspecific genetic diversity remain to be elucidated. Previous studies have examined certain aspects of the fundamental ecology of these species such as growth rates, mechanisms of seed dispersal and herbivore–plant interactions (Bullock & Heath, 2006; Franco‐Vizcaino et al., 2007; Wehncke & López‐Medellín, 2015; Wehncke, López‐Medellín, Wall, & Ezcurra, 2013; Wehncke et al., 2010). The geographic distributions of certain species based on morphological characteristics such as stem size, leaf shape and color, fruit shape and size, have also been described (Cornett 2008; Felger & Joyal, 1999; Oberbauer, 2005; Minnich et al., 2011). However, because morphological differences can sometimes be ambiguous and the majority of *Washingtonia* and *Brahea* populations on the peninsula are extremely hard to access (Felger & Joyal, 1999) no single study has effectively elucidated the distribution of all of the palms found in the Baja California Peninsula and Guadalupe Island.

In recent decades, molecular genetic studies have proven instrumental in resolving ongoing debates over the biogeographical origins of diverse taxa, including the Aracaceae (Baker & Couvreur, 2013; Couvreur et al., 2011; Kissling, Baker, et al., 2012, 2012; Kissling, Eiserhardt, et al., 2012, 2012; Kondo et al., 2012). However, the majority of studies of palms have focused on commercially important species (Barrett, Bacon, Antonelli, Cano, & Hofmann, 2016) and we are not aware of any published genetic studies of *Washingtonia* or *Brahea* species, with the exception of a single allozyme study of *W. filifera* in California (McClenaghan & Beauchamp, 1986).

Here we used *Washingtonia* and *Brahea* palms as a case study to explore how tropical species may have responded to the complex geological and ecological history of the mostly arid Baja California Peninsula and adjacent Guadalupe Island. These genera share similar life histories and habitat requirements yet both occupy the northern limit of the Arecaceae distributional range, are endemic and locally rare. To provide the first baseline genetic data on *Washingtonia* and *Brahea* palms from this region, we used a classical Sanger sequencing approach. By combining chloroplast and nuclear sequence data, we first set out to determine species delimitations on the Baja Peninsula. We then characterized patterns of haplotype diversity and distribution to address a number of questions: (i) Do sympatrically occurring *Washingtonia* and *Brahea* palms exhibit similar patterns of genetic structure along the Baja Peninsula? (ii) Is the restricted distribution of desert palms reflected in low genetic diversity and high divergence between major geographic regions? (iii) Are patterns of geographically structured genetic variation concordant with major historical vicariant events that shaped the region such as the formation of the Gulf of California, the inundation of the Isthmus of La Paz and the formation of Guadalupe Island?

## 2 MATERIALS AND METHODS

### 2.1 Sampling

**Figure 1:**
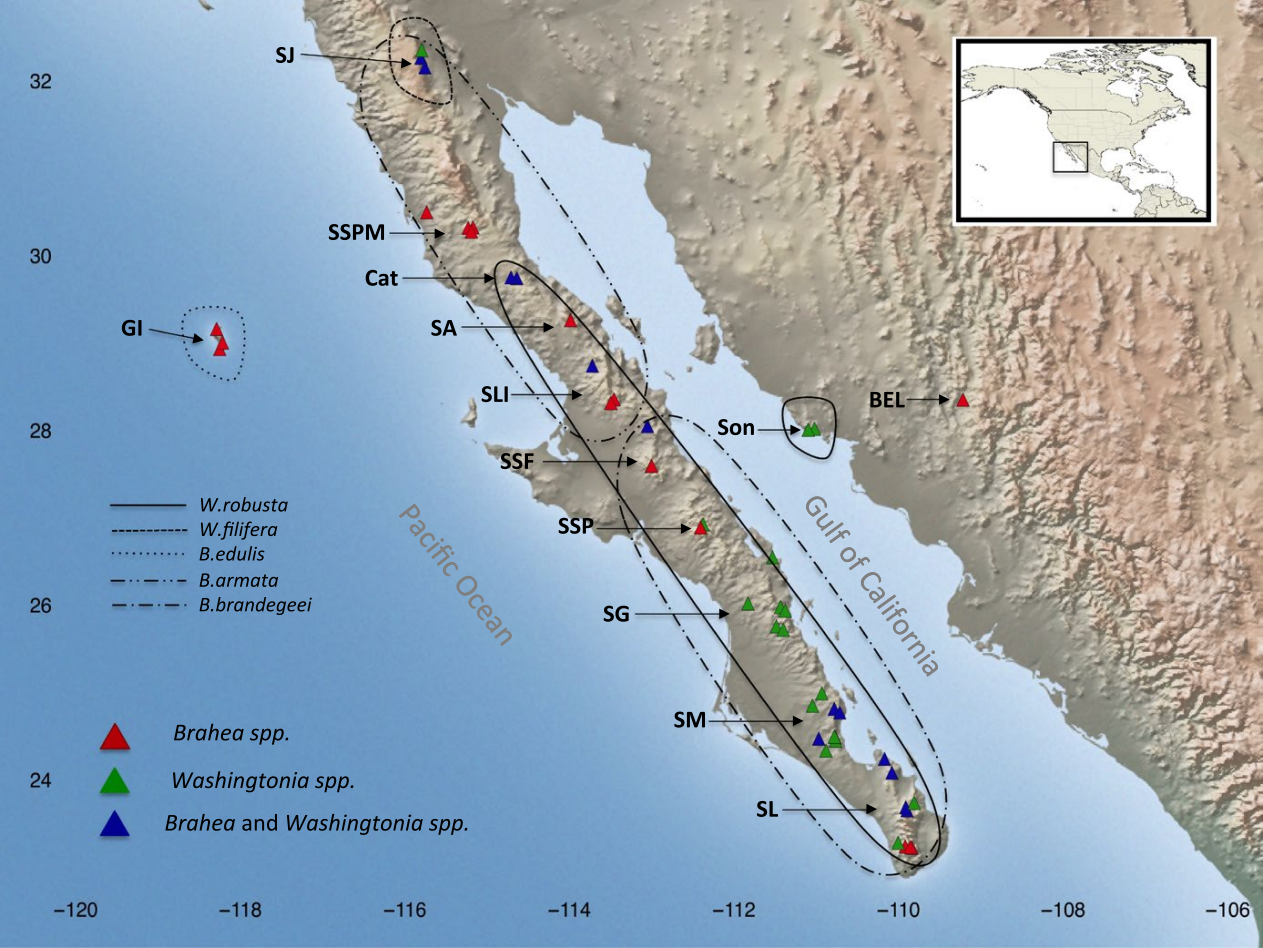
Map showing the sampling sites of *Washingtonia* spp. and *Brahea* spp. used in our study. The sampling and distributional limits of *W. filifera* were restricted only to the Mexican populations in Sierra Juarez. Abbreviations correspond to the names of the sampled sierras (populations): SL, Sierra La Laguna; SM, Sierra Mechudo; SG, Sierra Giganta; SSP, Sierra San Pedro; SSF, Sierra San Francisco; SLI, Sierra Libertad; SA, Sierra Asamblea; Cat, Cataviña; SSPM, Sierra San Pedro Martir; SJ, Sierra Juarez; GI, Guadalupe Island and SON, Mexican mainland

A total of 176 samples were collected from a total of five palm species (*Washingtonia* and *Brahea*) identified on the basis of their morphology in the field. Specimens were collected from virtually all accessible oases throughout the Baja California Peninsula as well as from Guadalupe Island and two sites in Sonora, Mexico (Figure 1). We avoided sampling immediately adjacent trees in order not to include close relatives in our dataset. Although our total sample size is modest, it is important to note that the species in question are rare and endemic, and normally grow in inaccessible canyons. Many sites could only be reached by foot or by riding a mule for several hours and in some cases days. Additionally, some stands were very small and were represented by only 5–10 individuals. The full distributional ranges of *W. robusta*,* B. armata*,* B. edulis,* and *B. brandegeei* were covered, although *W. filifera* was represented by samples only from Mexico. In total we collected 91 *Washingtonia* samples from eight populations (sierras), of which eight samples corresponded to *W. filifera* and 83 to *W. robusta*. For *Brahea,* we collected 85 samples from nine populations (sierras) and tenth popilation from Guadalupe Island, of which 17 samples belonged to *B. edulis*, 29 to *B. armata* and 39 to *B. brandegeei*. As some authors (e.g., Henderson et al., 1995) have argued that *B. brandegeei* may be synonymous to *B. elegans* from the Mexican mainland, we also included one herbarium specimen of this species. More details on sampled species, sierras and sites, and the number of amplified chloroplast and nuclear sequences can be found in Figure 1, Tables 1 and S1. The genetic data were generated from silica‐gel dried leaf samples collected in the field during 2015 and 2016.

**Table 1:** Numbers of *Washingtonia* and *Brahea* samples collected and successfully sequenced at nuclear and plastid loci. Also shown are the number of population sampled for each species and sampling sites are included for each population and species. The sampled populations (sierras) correspond to those shown in Figure 1

Species	Sierra (Population)	Number of sampling sites in each Sierra	Number of collected samples per population	Number of sequences amplified	
				Nuclear( )738 bp	Chloroplast 2,294 bp
*W robusta*	Cabo Region (SL)	5	16	16	11
	Sierra Mechudo (SM)	9	28	28	26
	Sierra La Giganta (SG)	5	12	12	12
	Sierra San Pedro (SSP)	1	4	4	3
	Sierra Aguaje (Son)	2	10	10	9
	San Fransisco de la Sierra (SSF) + Sierra Libertad (SLI)	2	8	7	7
	Cataviña (Cat)	2	5	5	5
*W. filifera*	Sierra Juárez (SJ)	3	8	7	8
Total *Washingtonia*		29	91	89	81
*B. brandegeei*	Cabo Region (SL)	7	18	18	17
	Sierra Mechudo (SM)	3	9	9	8
	Sierra San Pedro (SSP)	1	5	5	3
	San Fransisco de la Sierra (SSF)	2	7	6	7
*B. armata*	Sierra Libertad (SLI)	3	7	7	7
	SierraAsamblea (SA)	1	4	4	2
	Cataviña (Cat)	2	5	5	5
	Sierra San Pedro Mártir (SSPM)	4	7	5	7
	Sierra Juárez (SJ)	2	6	6	6
*B. edulis*	Guadalupe Island (GI)	3	17	14	17
*B. elegans*	Sonora (BEL)	1	1	1	1
Total *Brahea*		29	86	80	80

### 2.2 Molecular techniques

DNA extraction was performed using QIAGEN DNeasy plant mini kit (Valencia, CA, USA). As we are unaware of any population‐level genetic studies of *Washingtonia* or *Brahea*, we first evaluated three nuclear and seven chloroplast loci that have previously been used in phylogenetic and biogeographic studies of Arecaceae (Bacon, Baker, & Simmons, 2011; Bacon, Feltus, Paterson, & Bailey, 2008; CBOL Plant Working Group 2009; Jeanson, Labat, & Little, 2011; Shaw et al., 2005; Taberlet, Gielly, Pautou, & Bouvet, 1991). These loci (ITS 2, CISP 4, CISP 8, matK, rbcl, trnT‐trnD, trnG‐trnS, trnC‐rpoB, trnF‐trnL, trnfM‐trnS) were selected based on the amount of intrageneric diversity they possess in other plant species. For this trial, 40 samples of five palm species were PCR amplified in a 12.5 μl reactions containing 50–100 ng of DNA template, 1× PCR buffer, 1.5 mM MgCl2, 200 mM of dNTP mix, 0.4 mM of each primer, and 0.5 U of GoTaq Flexi DNA polymerase (Promega). The loci were amplified using primers and thermocycling protocols described elsewhere (Bacon et al., 2008; Jeanson et al., 2011; Shaw et al., 2005; Taberlet et al., 1991). Five loci (one nuclear and four chloroplast) were deemed suitable for further study and these were sequenced in all of the samples using the PCR conditions described above. The presence of amplified target DNA fragments was verified on 1.5% agarose gels stained with ethidium bromide. All positive PCR products were sent to Arizona GeneCore for sequencing in both directions. The resulting sequence chromatograms were assembled and manually edited using BioEdit 7.0.0 (Hall, 1999). The chloroplast sequences were then concatenated into a single sequence, as were the nuclear sequences, allowing subsequent analyses to be carried out separately for these two types of loci.

### 2.3 Molecular data analysis

Our sample size was somewhat restricted due to the difficulty of collecting samples from these locally rare and geographically restricted endemic species. We therefore tested if our final sample size was sufficient to recover most of the genetic diversity within the sampled populations. For this analysis, we constructed haplotype accumulation curves separately for the nuclear and chloroplast sequence data using the function haploAccum in the R package SPIDER (R Development Core Team 2008) with a total of 10,000 permutations. Molecular diversity statistics including the number of variable sites, the number of haplotypes, nucleotide and haplotype diversities, and the number of shared haplotypes were then computed using DnaSP 5.1 (Librado & Rozas, 2009) and ARLEQUIN 3.5.1.3 (Excoffier & Lischer, 2010). Phylogenetic relationships among the samples were inferred on the basis of the nuclear and chloroplast sequence data by constructing a parsimony network (Clement, Posada, & Crandall, 2000; Templeton, Crandall, & Sing, 1992) in PopArt (http://popart.otago.ac.nz, June 2016). We also used Gengis 2.1.1 (Parks et al., 2009) to visualize plastid and nuclear sequence geographic distributions.

To assess levels of genetic divergence between putative taxonomic species and *a priori* defined geographical regions (Figure 1, Table 1 and see below), we quantified pairwise differentiation using *F*_st_ (Weir & Cockerham, 1984) and Φ_st_ (Kimura, 1980). The former looks only at haplotype frequency differences (Excoffier, Smouse, & Quattro, 1992) while the latter also incorporates haplotype sequence similarity. *F*_st_ and Φ_st_ values were calculated within ARLEQUIN. For *Washingtonia*, eight geographic regions, each corresponding to a different sierra, were defined as follows: Mexican mainland (Son), Cabo Region (inluding Sierra La Laguna combined with Sierra Cacachilas, SL), Sierra del Mechudo (SM), Sierra la Giganta (SG), Sierra San Pedro (SSP), combined sites of Sierra Libertad and Sierra San Francisco (SLI + SSF), Cataviña (Cat) and Sierra Juárez (SJ). For *Brahea*, we specified ten geographical regions. Nine of these corresponded to different sierras on the Baja Peninsula: Cabo Region (SL), Sierra del Mechudo (SM), Sierra San Francisco (SSF), Sierra San Pedro (SSP), Sierra Libertad (SLI), Sierra Asamblea (SA), Cataviña (Cat), Sierra San Pedro Martir (SSPM) and Sierra Juárez (SJ); while the final geographical region corresponded to Guadalupe Island (GI). For further details including sampling coordinates, see Tables 1 and S1; Figures S1 and S2.

**Figure 2:**
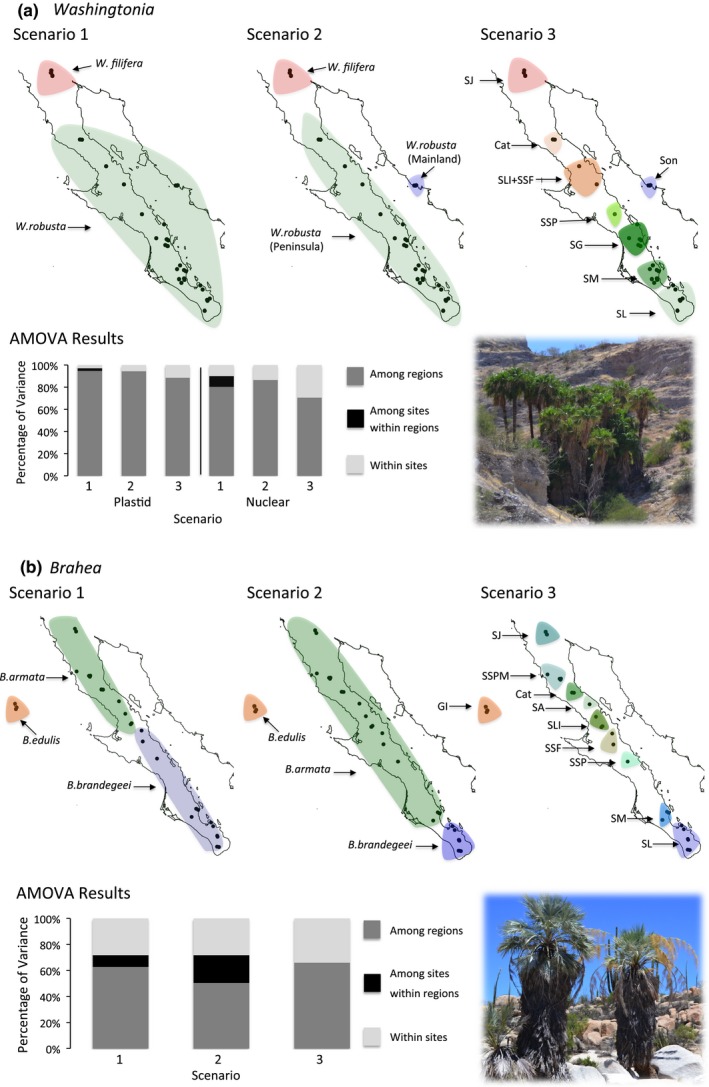
Panel (a). Upper panel: maps of the Baja peninsula and continental Sonora showing sampled *Washingotnia* populations. Each of the maps represents one hypothetical divergence scenario tested within AMOVA. The *a priori* defined regions are separated by three hypothesized phylogeographical breaks, including: (1) current taxonomy, (2) three major geographic regions and (3) each sierra as unique genetic entity. Arrows indicate species or sierras as follows: SL, Sierra Laguna; SM, Sierra Mechudo; SG, Sierra la Giganta; SSP, Sierra San Pedro; SLI + SSF, combined Sierra Libertad and Sierra San Francisco; Cat, Cataviña; SJ, Sierra Juarez; and Son, Sierra Aguaje. Lower left panel: results of analysis of molecular variance (AMOVA) comparison of three vicariance scenarios, based on nuclear and plastid markers. Lower right panel: picture of one of the sampling sites of *W. robusta* in Sierra Mechudo (SM). Panel (b). Upper panel: maps of the Baja peninsula and Guadalupe Island showing sampled *Brahea* populations. Each of the maps represents one hypothetical divergence scenario tested with AMOVA. The *a priori* regions are separated by three hypothesized phylogeographical breaks, including (1) current taxonomy, (2) isolation of Cabo region after oceanic inundation of the Isthmus of La Paz, and (3) each sierra as unique genetic entity. Arrows indicate species or sierras as follows: SL, Sierra Laguna; SM, Sierra Mechudo; SSP, Sierra San Pedro; SSF, Sierra San Francisco; SLI, Sierra Libertad; SA, Sierra Asamblea; Cat, Cataviña; SSPM, Sierra San Pedro Martir; SJ, Sierra Juarez; and final tenth group GI, Guadalupe Island. Lower left panel: results of analysis of molecular variance (AMOVA) comparison of three vicariance scenarios, based on nuclear markers. Lower right panel: picture of one of the sampling sites of *B. armata* in Sierra Asamblea (SA)

We also used hierarchical analysis of molecular variance (AMOVA) to partition the genetic variation within *Washingtonia* and *Brahea* using ARLEQUIN. We used AMOVA to estimate relative support for three alternative scenarios (Figure 2). Each scenario specified a hierarchical partitioning of populations within regions/groups, and the nesting scheme that maximized the among‐group variance component was considered the best divergence scenario. The first scenario was based on the current taxonomical delimitation of the species (i.e. two groups were specified for *Washingtonia*, corresponding to *W. filifera* and *W. robusta* and three groups were specified for *Brahea* corresponding to *B. edulis*,* B. armata,* and B. *brandegeei*). Second, for the *Washingtonia* palms, we constructed a scenario based on the isolation of the mainland and peninsular populations after the formation of Sea of Cortez into three major regions: the Mexican mainland, Baja Peninsula and samples of *W. filifera*. Third, for the *Brahea* palms, we partitioned all of the samples into three groups: Guadalupe Island, Cabo region and rest of the Baja Peninsula. Fourth, for both *Washingtonia* and *Brahea* palms, we tested the hypothesis that relict populations persisted within each of the sierras, which should be reflected in each sierra harboring a genetically distinctive population. Eight groups were specified for *Washingtonia* and ten for *Brahea*, each corresponding to a different sierra (For more details see Figure 2). AMOVA and genetic differentiation analyses were not carried out for the *Brahea* chloroplast data, as only three haplotypes were present and almost all of the individuals shared the same haplotype. The single *B. elegans* sample was also excluded from these analyses. For each AMOVA, statistical significance was determined using 10,000 permutations of the dataset.

Finally, we used a Bayesian approach to estimate the number of genetic clusters present within the data as implemented in BAPS 6 (Corander, Marttinen, Siren, & Tang, 2008; Corander, Siren, & Arjas, 2008; Corander, Waldmann, & Sillanpaa, 2003). For this analysis, individuals were first clustered without any previous knowledge of their sampling locations. This analysis was run using the mixture model to determine the most probable number of clusters (*K*) within the data. *K* was set from 1 to 10 and five replicates were performed for each value of *K*. An admixture analysis was then carried out with 50 reference individuals and 1,000 iterations. Finally, we spatially clustered the individuals using Voronoi tessellation and geo‐referenced sample location data. All of the spatial analyses were conducted separately for *Washingtonia* and *Brahea*.

### 2.4 Ecological niche modeling

To better understand the current and past population dynamics of *Washingtonia* and *Brahea* palms along the Baja Peninsula we performed Ecological Niche Modeling (ENM) using the maximum entropy method implemented in Maxent 3.4.0 (Phillips, 2017; Phillips, Anderson, & Schapire, 2006). Maxent attempts to estimate a probability distribution of species occurrence that is closest to uniform while still subject to environmental constraints (Elith et al., 2011). Information on the geographic distribution of *Washingtonia* and *Brahea* was based on a total of 49 and 44 unique presence records respectively. We combined our field data, information of natural occurrence of *Washingtonia* in the USA described in Cornett et al. (1986) and geo‐referenced occurrence data from www.gbif.org and www.bajaflora.org (Accessed March 2017). Each model included seven bioclimatic variables that we considered might have the strongest effect on the distribution of these species (mean temperature of wettest quarter, mean temperature of driest quarter, mean temperature of warmest quarter, mean temperature of coldest quarter, precipitation of wettest quarter, precipitation of driest quarter, and precipitation of coldest quarter). By restricting this analysis to a subset of the most promising variables, we avoided potential overfitting of the Maxent model (Peterson & Nakazawa, 2008). To gain a better perspective on temporal changes in the distributions of *Washingotnia* and *Brahea* palms, we predicted suitable climate envelopes for each genus using current ecological conditions (1960–1990) and the following three historical time periods: mid Holocene (~6,000 years BP), the last glacial maximum (~22,000 years BP) and the last interglacial (~130,000 years BP). All of the bioclimatic data were downloaded from WorldClim (www.worldclim.org) as a set of raster layers with a spatial resolution of 30 s (ca. 1 km) or in the case of the LGM data, 2.5 min (ca. 5 km) (Hijmans, Cameron, Parra, Jones, & Jarvis, 2005; Otto‐Bliesner, Marshall, Overpeck, Miller, & Hu, 2006; Watanabe et al., 2011). For each run, we used the default settings with 30 bootstrap replicates. The area under the receiver operating curve (AUC) was calculated to assess the model accuracy, with values between 0.7 and 0.9 indicating good discrimination (Swets, 1988). Jackknife analyses were performed to determine the relative contribution of each of environmental variable to the model of projected current distribution (Phillips, 2017).

In order to test the null hypothesis that the two Baja palm's species occupy identical climatic environments (“niches”), we performed a niche identity test using ENMtools 1.4.4 (Warren, Glor, & Turelli, 2010) with 1,000 pseudoreplicates. We then compared a distribution of niche similarities obtained from pairs of pseudoniches based on randomly sampled occurrence points with the actual niche overlap between *Washingtonia* and *Brahea* (Warren et al., 2010). The latter was quantified using Schoener's *D* (Schoener, 1968) and the standardized Hellinger distance (*I*) (Warren, Glor, & Turelli, 2008). Schoener's *D* assumes that the suitability scores are proportional to species abundance, whereas Hellinger's‐based *I* quantifies the probability distributions of two ecological niche models. Both similarity metrics range from 0 (no niche overlap) to 1 (identical niches) (Warren et al., 2010).

## 3 RESULTS

### 3.1 Molecular markers

We evaluated a total of ten chloroplast and nuclear loci that have been proposed for molecular studies of the Arecaceae (Bacon et al., 2008; CBOL Plant Working Group 2009; Jeanson et al., 2011). Of these, we selected five informative loci: matK, CISP 4, trnT‐trnD, trnG‐trnS, and trnF‐trnL for further analysis. The other loci either lacked sequence variation in a representative sample of individuals (rbcL and trnC‐rpoB), failed to PCR amplify (trnfM‐trnS) or amplified more than one PCR product (ITS2 and CISP 8).

### 3.2 Sequence diversity

**Figure 3:**
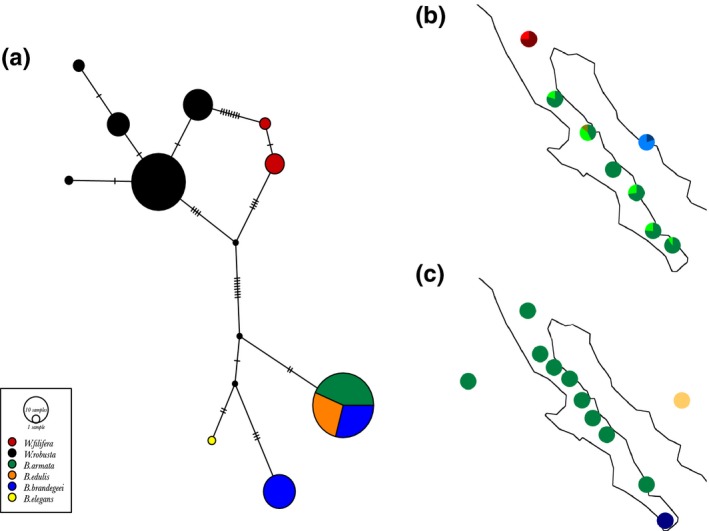
Geographical distribution of *Washingtonia* and *Brahea* chloroplast haplotypes. Panel (a) shows a haplotype network in which the size of each pie chart is proportional to the number of individuals carrying the haplotype. Each tick mark on the solid lines indicates a single DNA mutation. The colors shown in the box correspond to species delineations based on morphological characters as described in Minnich et al. (2011). Geographical distributions of chloroplast haplotypes of (b) *Washingtonia* and (c) *Brahea* samples. Colors correspond to the different haplotypes

**Figure 4:**
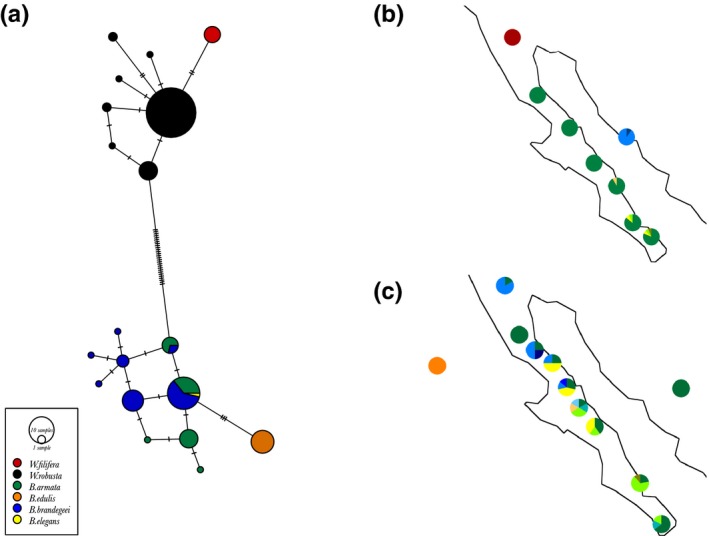
Geographical distribution of *Washingtonia* and *Brahea* nuclear haplotypes. Panel (a) shows a haplotype network in which the size of each pie chart is proportional to the number of individuals carrying the haplotype. Each tick mark on the solid lines indicates a single DNA mutation. The colors shown in the box correspond to species delineations based on morphological characters as described in Minnich et al. (2011). Geographical distributions of nuclear haplotypes of (b) *Washingtonia* and (c) *Brahea* samples. Colors correspond to the different haplotypes

The final data set included 2,294 bp of concatenated chloroplast sequence and 738 bp of concatenated nuclear sequence, from which all InDels had been removed (All DNA sequences newly generated in this study have been deposited in the GenBank database under Accession Numbers KY911112‐KY911153). In total, we obtained 89 nuclear and 81 plastid sequences for *Washingtonia* palms and 80 nuclear and 80 plastid sequences from *Brahea* palms, including a single sample of *B. elegans* from Mexican mainland (See Section 2 and Table 1 for more details). Pooling all of the samples, we obtained a total of 10 unique chloroplast and 19 nuclear haplotypes (Table 2, Figures 3 and 4). Levels of sequence diversity were generally low within the morphologically defined species, with individuals carrying either a single common haplotype or a handful of closely related haplotypes at low frequency (Table 2). As shown by the haplotype accumulation curves, our sample size was sufficiently large to encompass the majority of the genetic diversity present within the studied species (Figure S1).

**Table 2:** Descriptive statistics for (a) chloroplast and (b) nuclear sequences isolated from five desert palm species

		*W. robusta*	*W. filifera*	*B. armata*	*B. brandegeei*	*B. edulis*
(a) Chloroplast	*n* samples	73	8	27	35	17
	*n* haplotypes	5	2	1	2	1
	Variable sites	4	1	–	6	–
	*h*	0.53	0.43	–	0.51	–
	π	0.0003	0.0002	–	0.001	–
(b) Nuclear	*n* samples	82	7	27	38	14
	*n* haplotypes	7	1	5	7	1
	Variable sites	6	–	4	5	–
	*h*	0.34	–	0.72	0.70	–
	π	0.0006	–	0.0013	0.0014	–

*h* haplotype diversity and π nucleotide diversity. We excluded *B. elegans* from these analyses as it was represented only by one sample.

### 3.3 Relationships among and within species

Parsimony networks were used to visualize relationships among the samples based on the concatenated chloroplast and nuclear sequences (Figures 3a and 4a respectively). As expected, *Washingtonia* and *Brahea* were deeply divergent from one another at both the chloroplast and nuclear DNA. Moreover, within the genus *Washingtonia*,* W. robusta,* and *W. filifera* differed by nine (chloroplast) and two (nuclear) fixed nucleotides and carried no shared haplotypes (Figures 3a and 4a). Accordingly, when we plotted the spatial distributions of the chloroplast and nuclear haplotypes, *Washingtonia* samples formed three distinct groups with no shared haplotypes among them (Figures 3b and 4b). These comprised a northern group of *Washingtonia* from the Sierra Juarez (SJ), a group from the Baja California Peninsula and a group of mainland populations.

Within the *Brahea* complex, contrasting results were obtained for the plastid and nuclear sequence data (Figures 3a,c and 4a,c). Despite extensive sampling and having sequenced over 2,000 bp of chloroplast DNA, all of the *B. armata*,* B. edulis,* and northern *B. brandegeei* samples were fixed for a single haplotype (Figure 3a,c) and thus could not be distinguished from one another. The single *B. elegans* sample that was collected from the Mexican mainland carried a unique chloroplast haplotype separated by five mutational steps from *B. armata* and *B. edulis*. *B. brandegeei* was represented by two haplotypes, one of which was shared with *B. armata* and *B. edulis*, whereas another haplotype was only found in samples from the SL population (Figure 3c). Correspondingly greater haplotype diversity was found at the nuclear sequence data, with a total of 11 haplotypes detected, although most of these were present at low frequencies. There was also considerably more haplotype sharing among the *Brahea* species, with only *B. edulis* being distinguished by a unique nuclear haplotype (Figure 4a). Spatial distributions of the nuclear haplotypes for *Brahea* did not reveal any clear geographical patterns along the Peninsula, but confirmed the uniqueness of Guadalupe Island palms (Figure 4c).

Pairwise differentiation statistics provided additional quantitative support for the patterns described above (Tables 3 and 4, Table S2). For example, within *Washingtonia* and for both the chloroplast and nuclear data, pairwise *F*_st_ and Φ_st_ values were large and highly significant for the comparisons between *W. filifera* (SJ) and the peninsular and mainland *W. robusta* populations. In contrast, none of the comparisons among the sierras of the Baja Peninsula were significant (Table 3a,b).

**Table 3:** Estimates of genetic differentiation among eight populations of *Washingtonia*. Pairwise *F*_st_ and Φ_*st*_ values (above and below the diagonal respectively) are shown separately for (a) chloroplast and (b) nuclear genes

	Sierra (Population)	SJ	Cat	SLI + SSF	SSP	SG	SM	SL	Son
(a) Chloroplast	SJ	–	**0.97** ***	**0.96** ***	**0.98** **	**0.97** ***	**0.97** ***	**0.98** ***	**0.97** ***
	Cat	**0.58** ***	–	−0.15	−0.13	−0.12	−0.13	−0.11	**0.74** ***
	SLI + SSF	**0.46** **	−0.11	–	−0.06	−0.08	−0.04	0.03	**0.68** ***
	SSP	**0.69** **	−0.13	0.05	–	0.06	−0.02	−0.19	**0.77** **
	SG	**0.54** ***	−0.12	−0.09	0.06	–	−0.04	0.08	**0.72** ***
	SM	**0.61** ***	−0.13	−0.03	−0.02	−0.04	–	0.00	**0.74** ***
	SL	**0.71** ***	−0.11	0.11	−0.19	0.08	0.00	–	**0.79** ***
	Son	**0.61** ***	**0.63** ***	**0.51** ***	**0.73** **	**0.58** ***	**0.63** ***	**0.74** ***	–
(b) Nuclear	SJ	–	**1.00** **	**1.00** ***	**1.00** **	**0.91** ***	**0.92** ***	**0.85** ***	**0.96** ***
	Cat	**1.00** **	–	0.00	0.00	−0.10	−0.11	−0.10	**0.87** ***
	SLI + SSF	**1.00** **	0.00	–	0.00	−0.06	−0.07	−0.06	**0.89** ***
	SSP	**1.00** **	0.00	0.00	–	−0.13	−0.14	−0.14	**0.86** **
	SG	**0.81** ***	−0.06	−0.02	−0.10	–	0.01	0.00	**0.76** ***
	SM	**0.89** ***	−0.09	−0.06	−0.13	0.00	–	−0.03	**0.82** ***
	SL	**0.77** ***	−0.05	−0.01	−0.09	−0.03	−0.01	–	**0.72** ***
	Son	**0.87** ***	**0.86** **	**0.87** ***	**0.84** **	**0.72** ***	**0.83** ***	**0.71** ***	–

FDR corrected *p*‐values: *p < .05; **p < .01; ***p < .001, *p*‐values at α < 0.05 are highlighted in bold. The coding corresponds to the sampled sierras (See Section 2, Figure 1 and Table 1 for more details).

**Table 4:** Estimates of genetic differentiation among the ten populations of *Brahea* based on the nuclear data. Pairwise *F*_st_ and Φ_*st*_ values are shown above and below the diagonal, respectively

Sierra (Population)	SJ	SSPM	Cat	SA	SLI	SSF	SSP	SM	SL	GI
SJ	–	**0.78** *	−0.09	0.41	0.29	**0.61** *	**0.55** *	**0.66** **	**0.53** **	**0.97** ***
SSPM	**0.78** *	–	0.55	0.29	0.16	**0.49** *	0.17	**0.49** *	0.09	**1** **
Cat	−0.04	0.50	–	0.21	0.17	**0.49** *	0.38	**0.55** *	**0.45** *	**0.95** **
SA	0.27	0.50	−0.03	–	−0.21	0.26	−0.14	**0.42** *	0.16	**0.94** **
SLI	0.30	0.37	0.04	−0.21	–	0.21	−0.11	**0.31** *	0.09	**0.86** ***
SSF	**0.35** *	**0.40** *	0.07	0.07	0.09	–	0.14	0.05	**0.24** *	**0.89** **
SSP	**0.41** *	0.33	0.09	−0.17	−0.13	−0.00	–	0.25	−0.04	**0.93** ***
SM	**0.52** **	**0.56** *	0.30	0.30	**0.28** *	0.01	0.15	–	0.16	**0.92** ***
SL	**0.48** **	0.06	**0.24** *	**0.24** *	**0.20** *	0.14	0.09	**0.27** *	–	**0.88** ***
GI	**0.90** ***	**1** ***	**0.81** ***	**0.81** ***	**0.71** ***	**0.69** ***	**0.77** ***	**0.77** ***	**0.71** ***	–

FDR corrected *p*‐values: *p < .05; **p < .01; ***p < .001, *p*‐values at α < 0.05 are highlighted in bold. The coding corresponds to the sampled sierras (See Section 2, Figure 1 and Table 1 for more details).

Within *Brahea*, we focused on the nuclear data, as only three chloroplast haplotypes were present, one of which was found in 78% of the samples. For the nuclear sequence data, *Brahea* samples were grouped into ten *a priori* defined geographical regions (See Section 2, Table 1 and Figures 1 and 2 for more details). The resulting *F*_st_ and Φ_st_ values between Guadalupe Island and the other regions ranged from 0.69 to 1 and were thus highly significant (Table 4). We also detected significant differentiation between the southern (Cabo Region, Sierra del Mechudo, Sierra San Pedro) and northern (Sierra Juárez, Sierra Libertad and Cataviña) sierras (Table 4). After partitioning the samples based on taxonomically designated species, similarly high and significant *F*_st_ and Φ_st_ values were obtained (Table S2).

Next, we used AMOVA to assess how sequence variation was partitioned among species and *a priori* defined geographical regions (Figure 2 and Table S3a,b). All partitioning schemes yielded significant estimates of among‐region differentiation. In the case of *Washingtonia* palms, of the three hypotheses that we tested, the grouping of the samples into three regions corresponding to *W. robusta* from the mainland, *W. robusta* from the Baja Peninsula and *W. filifera* yielded the largest among‐region variance components for nuclear (87%) and plastid (94%) data (Scenario 2 in Figure 2a). For *Brahea* palms, the proportion of variance attributable to differences among the ten sierras was greatest, at around 66% (Scenario 3 in Figure 2b), followed by among taxonomically designated species (62%) and *B. brandegeei* being restricted to SL (50%) (Table S4).

### 3.4 Bayesian cluster analysis

The above results were strongly supported by Bayesian cluster analyses of the combined chloroplast and nuclear sequence datasets conducted within BAPS. Without utilizing prior information on the sampling locations of individual palms, analysis of the chloroplast data revealed support for four genetic clusters (*K* = 4, Figure S2a). Two clusters were found within *Washingtonia*, corresponding to the taxonomically defined *W. filifera* and *W. robusta*. A further two clusters were found within *Brahea*, corresponding to *B. brandegeei* samples from the SL population plus the single sample of *B. elegans* from the mainland, and *B. edulis* plus all of the *B. brandegeei* samples north of the Cabo region and all of the *B. armata* individuals. Based on the nuclear data, only three clusters were identified (*K* = 3, Figure S2b). Specifically, all of the *Washingtonia* samples clustered together, as did all of the *Brahea* samples with the exception of *B. edulis* from Guadalupe Island, which formed a third cluster.

**Figure 5:**
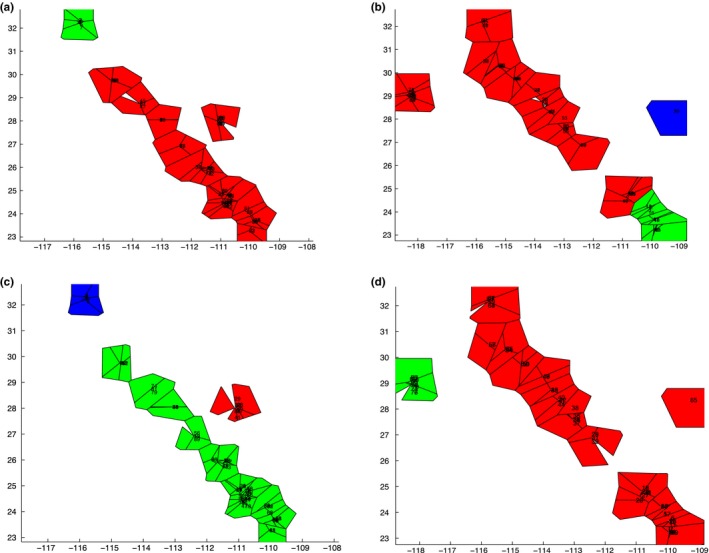
Spatial clustering analysis of *Washingtonia* and *Brahea* samples using a Voronoi tessellation as implemented in BAPS. (a) *Washingtonia* spp. chloroplast data, (b) *Brahea* spp. chloroplast data, (c) *Washingtonia* spp. nuclear data, and (d) *Brahea* spp. nuclear data

Finally, we used information on the sampling sites to conduct spatial clustering analyses within BAPS separately for *Washingtonia* and *Brahea* based on the chloroplast and nuclear data respectively (Figure 5). For *Washingonia*, Voronoi tessellation graphs of both the chloroplast (Figure 5a) and nuclear (Figure 5c) sequence data revealed two geographically separate genetic clusters, corresponding to *W. filifera* in the north (SJ) and *W. robusta* throughout the rest of the Baja Peninsula. Based on the nuclear data, an additional cluster was recovered corresponding to samples from the mainland *Washingtonia* populations (Figure 5c). For *Brahea*, the chloroplast data revealed three clusters (Figure 5b) that exactly mirror the spatial haplotype distributions shown in Figure 2c. By contrast, the nuclear data indicated the presence of only two genetic clusters (Figure 5d), one corresponding to the peninsular and mainland populations combined, and the second to Guadalupe Island.

### 3.5 Ecological niche modeling

AUC values of the ecological niche models of projected current distributions, averaged over 30 replicates, were around 0.97 for both *Washingtonia* and *Brahea* indicating good predictive model performance. For *Washingtonia*, the environmental variables that contributed the most to the Maxent model of projected current distribution were precipitation of the driest quarter, mean temperature of the coldest quarter and mean temperature of the wettest quarter in ascending order. Together these variables contributed over 70% of the explanatory power of the model, indicating that they are very good predictors of the contemporary geographical distribution of *Washingtonia* palms. For *Brahea*, precipitation of driest quarter, mean temperature of the warmest quarter and mean temperature of the driest quarter were the main predictors of geographical distribution, contributing over 67% of the explanatory power of the model (Table S5).

**Figure 6:**
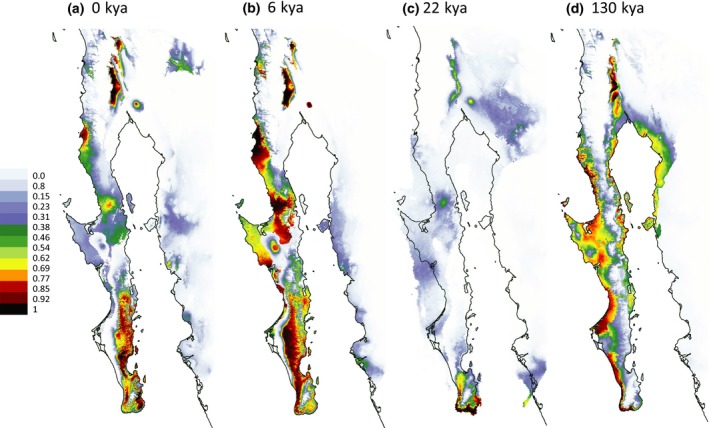
Probability of occurrence of *Washingtonia palms* on the Baja California: (a**)** at present; (b) during the mid Holocene; (c) during the last glacial maximum and (d) during the last interglacial. See the main text for more details

**Figure 7:**
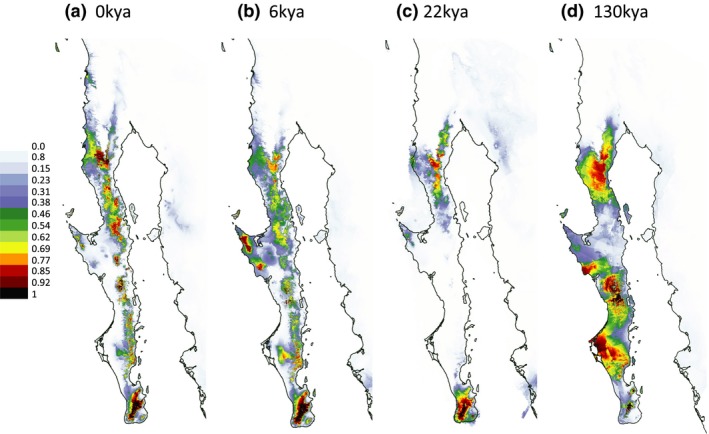
Probability of occurrence of *Brahea palms* on the Baja California: (a**)** at present; (b) during the mid Holocene; (c) during the last glacial maximum, and (d) during the last interglacial. See the main text for more details

In general, the predicted distribution of *Washingtonia* under current conditions (Figure 6A) was similar to the actual distribution (Minnich et al., 2011 and Figure 1), with two major areas of high probability of occurrence, one in the southern Baja Peninsula (SL, SM and SG) and the other in the northern Baja Peninsula (SJ) and southern California. However, there were also some predicted areas where *Washingtonia* palms cannot currently be found, such as the Pacific coast around the 30th parallel north. The projected current distribution of *Brahea* was somewhat restricted in comparison to *Washingtonia*, with SL, SSP and SSPM being the main areas of high probability of occurrence (Figure 7a).

During the mid Holocene (about 6,000 years ago) the predicted geographical distribution of *Washingtonia* was broader than the contemporary distribution, with favorable habitats situated especially along the Pacific coast of the central and southern Baja Peninsula (Figure 6b). By contrast, the predicted distribution of *Brahea* during the mid Holocene was similar to its current projected distribution (Figure 7b). Further back in time, during the last glacial maximum (about 22,000 years ago), both species appear to have been restricted largely to the southernmost tip of the peninsula, with *Brahea* also predicted to occur in a small area of the mid‐northern peninsula (Figures 6c and 7c). According to Maxent estimations, during the last interglacial (about 130,000 years ago), *Washingtonia*'s predicted distribution shifted mainly to coastal areas of both the northern and southern peninsula (Figure 6d). *Brahea* on the other hand seems to had have experienced favorable conditions across much of the peninsula, which extended into SG, SSP and SSPM (Figure 7d). The estimated niche overlap for *Washingtonia* and *Brahea* was considerable of 0.55 and 0.7 using Schoener's *D* and Hellinger's‐based *I,* respectively. Nonetheless, randomization tests of niche identity indicated that *Washingtonia* and *Brahea* palms are not ecologically equivalent to each other (Figure S3), regardless of the measure of similarity used (Schoener's *D* or Hellinger's *I*).

## 4 DISCUSSION

We have generated baseline molecular data on species boundaries and the population structure of native palms of Baja California and Guadalupe Island, which provide new insights into the histories of these unique species. Our initial prediction regarding the levels of genetic diversity and phylogeography of *Washingtonia* and *Brahea* were only partially confirmed. In accordance with expectations, disjunct and isolated populations of Baja palms contained low haplotype diversity. Additionally, ecological barriers like sea channels appear to be responsible for high levels of isolation between groups on either side of the barrier. In contrast to expectation however, genetic structure and diversity distribution of *Washingtonia* and *Brahea* along the Peninsula were not the same. Detailed ecological niche modeling analysis provided an explanation for this trend, suggesting slightly different re‐colonization routes after the LGM and sensitivity to different ecological variables. Our work demonstrates the importance and advantages of a multispecies approach, as some relevant conclusions about each species can only be reached using a comparison among different, yet similar and related species.

### 4.1 Species delimitation

One of the main goals of our work was to establish species boundaries for *Washingtonia* and *Brahea* palms in the study area. The distribution of *Washingtonia* inferred from our sequence data is broadly in line with previously published morphological information (Felger & Joyal, 1999; Minnich et al., 2011). Despite alleged hybrids having been reported on the basis of morphological data at Cataviña (Cornett, 1987b) and hybridization between *W. robusta* and *W. filifera* being common under cultivation (Hodel, 2014), we found no shared haplotypes between *W. robusta* and *W. filifera* and the distributional limits of these two species inferred from the sequence data also did not overlap, the southern limit of *W. filifera* being Sierra Juarez and the northern limit of *W. robusta* being Cataviña (Figures 3 and 4). Overall, given the lack of shared haplotypes and strong sequence divergence between *W. filifera* and *W. robusta*, our data support the classification of these two taxa as separate, geographically delimited, species.

In contrast, our sequence data revealed mixed support for previous morphological species definitions within *Brahea*. For instance, Minnich et al. (2011) recently suggested that *B. brandegeei* is distributed on the southern part of the peninsula from the Sierra La Laguna to the Sierra San Francisco. However, this was not supported by either the nuclear or the chloroplast data, the former showing no obvious split in the genetic composition of *Brahea* populations along the entire length of the peninsula, while the latter revealed a lineage corresponding to *B. brandegeei* that appears to be restricted to the Cabo region (SL) of the Peninsula. Our study also included *B. edulis* samples from Guadalupe Island, allowing a comparison to be made with the peninsular *Brahea* populations. We found that *B. edulis* was strongly differentiated from the peninsular populations at the nuclear sequence data, being fixed for a unique haplotype, but that at the chloroplast sequence data *B. edulis* could not be distinguished from the northern peninsula populations corresponding to *B. armata*.

There are several possible explanations for the observed differences between the chloroplast and nuclear sequence data within *Brahea*. First, introgression of chloroplast DNA cannot be discounted, but this seems unlikely given the degree of geographic isolation of Guadalupe Island and its geological origin (Moran, 1996). Second, incomplete linage sorting of shared ancestral polymorphisms could be important, especially given the relatively low levels of observed sequence polymorphism, which could reflect slow chloroplast mutation rates in palms (Wilson, Gaut, & Clegg, 1990). Third, the chloroplast genome reflects seed‐mediated gene flow (Hamrick & Nason, 2000) suggesting that a contrasting pattern at the two types of marker could arise if partial geographical isolation interrupted seed‐mediated gene flow but not pollen‐mediated genetic exchange (Hamilton & Miller, 2002; Petit et al., 2005). Regardless of the exact explanation, incongruent phylogenetic signals between plastid and nuclear data are not uncommon in plants (Pillon et al., 2013), suggesting that an interesting avenue for further research in palms would be to develop a larger and more informative marker panels, for instance using new approaches such as genotyping by sequencing (Narum, Buerkle, Davey, Miller, & Hohenlohe, 2013).

### 4.2 Patterns of haplotype diversity and structure

Although we expected that sympatrically occurring *Washingtonia* and *Brahea* palms would exhibit similar patterns of haplotype structuring along the Baja Peninsula we found the opposite (Figures 3 and 4). For example, as inferred with AMOVA and BAPS, *Washingtonia* palms on the Baja Peninsula were clustered into two groups comprising palms from SJ and the rest of the peninsula. Contrastingly, AMOVA analysis of the nuclear sequences in *Brahea* favored a scenario in which each sierra was genetically distinct (Figure 2b, Scenario 3). These differences may be explained by the slightly distinct habitat requirements and differences in the drought and freezing tolerances of *Washingtonia* and *Brahea* (Cornett, 1987a,b; Franco‐Vizcaino et al., 2007; Minnich et al., 2011). In accordance, ENM indicated that although the strongest ecological predictor of the contemporary distribution of *Washingtonia* and *Brahea* palms was precipitation of driest quarter, the second most important variable affecting *Washingtonia's* distribution was the mean temperature of the coldest quarter, whereas for *Brahea* it was the mean temperature of the warmest quarter. Predicted temporal changes in the distribution of these two genera over the past 130,000 years also indicated that *Washingotnia* and *Brahea* probably responded differently to historical changes in climate (Figures 6 and 7). For example, the contemporary distribution of *Washingtonia* suggests that its range may have contracted over recent historical time whereas *Brahea* has maintained a similar distribution for at least 6,000 years.

Dispersal is an essential element that shapes patterns of genetic structure in the Arecaceae (Eiserhardt, Svenning, Kissling, & Balslev, 2011). Although many vertebrates like bats, coyotes and foxes have been proposed as possible seed dispersal agents for *Washingtonia* and *Brahea*, the effect of these agents on palm genetic diversity and structure has not been properly tested (Cornett, 2008; Wehncke et al., 2010). In the case of *Brahea,* it also appears that water pulses may be more significant in terms of seed dispersal than vertebrates (Wehncke et al. 2010; Wehncke, López‐Medellín, & Ezcurra, 2009). A comparative study that would quantify the differences in dispersal capacity between *Washingtonia* and *Brahea* would definitively help to further our understanding of this topic.

As expected, levels of haplotype diversity were low in *Washingtonia* for both the chloroplast and nuclear sequence data, whereas nuclear diversity in peninsular *Brahea* was somewhat higher (Figures 3 and 4). The levels of diversity that we found were broadly comparable to other relict plant species of the region (e.g., *Guaiacum unijugum*, McCauley et al., 2010; *Quercus brandegeei,* Cavender‐Bares et al., 2015) as well as to *Washingtonia* populations in the USA (McClenaghan & Beauchamp, 1986). Moreover, sequence diversity at the nuclear loci was not evenly distributed along the peninsula and also showed differences between *Washingtonia* and *Brahea* (Figure 4). In *Washingtonia* palms, higher diversity was found in the southern sierras (SL and SM), whereas in *Brahea* palms, the mid peninsular sierras (SSF and SLI) had the highest nuclear sequence diversity (Figure 4). Higher levels of diversity in the southern populations of *Washingtonia* may be explained by postglacial range expansion and re‐colonization of the northern peninsula from southern refugia, as has been suggested for other Baja Peninsula plant species (Clark‐Tapia & Molina‐Freaner, 2003; Gutiérrez Flores, García‐De León, León de la Luz, & Cota‐Sánchez, 2016; Nason, Hamrick, & Fleming, 2002). This scenario was also supported by ENM analysis (Figure 6c), which suggested that during the LGM, predicted suitable habitats for *Washingtonia* became restricted to a narrow coastal strip at the southern tip of the Baja Peninsula. Interestingly, another predicted habitat where *Washingtonia* was likely to survive was in southern California. Nonetheless, based on the observed haplotype distribution, palms from this refugium were able to re‐colonize only SJ and did not move further south. This may be explained by the observation that Sierra San Pedro Martir has acted as a natural dispersal barrier for *Washingtonia* (Minnich et al., 2011); ENM results also supported this explanation (Figure 6). However, *W. robusta* is naturally rare in the northern peninsula, has higher water requirements and seems to be less tolerant to low temperatures than *Brahea* (Minnich et al., 2011). We therefore cannot discount the possibility that these observed patterns could have been partly affected by selection favouring the retention of specific haplotypes through linkage disequilibrium to functional loci. One way to test for this would be to genotype functional genetic markers putatively involved in adaptation (Luikart, England, Tallmon, Jordan, & Taberlet, 2003).

The pattern of sequence diversity found in *Brahea* may be explained by the presence of refugia not in the south, but in the middle part of the peninsula (Garrick, Nason, Meadows, & Dyer, 2009). However, ENM analysis contradicted this explanation and showed that the predicted distribution of *Brahea* during the LGM was concentrated across relatively large areas in SL and SSPM (Figure 7). Another possibility that may explain the high genetic diversity of *Brahea* palms in the middle of the peninsula would be that this region represents the contact zone of recolonization between palms of the southern (SL) and northern (SSPM) refugia during the LGM. This hypothesis would also help to explain why it has been hard to established precise species boundaries between *B. brandegeei* from the southern and *B. armata* from the northern peninsula using morphological characteristics (Felger & Joyal, 1999; Minnich et al., 2011).

Moving to the Guadalupe Island palms, we found comparably much lower levels of chloroplast and nuclear diversity. Only one chloroplast and one nuclear haplotype was recovered for 17 and 14 *B. edulis* individuals respectively. This pattern is in contrast to results found for other tree species of the Island (e.g., *Pinus radiata*, Karhu, Vogl, Moran, Bell, and Savolainen (2005) and *Cupressus guadalupensis*, Rosas‐Escobar et al. (2011). Although founder effects are often invoked to explain low levels of genetic variation, many other factors may influence the genetic diversity of endemic island plants, including extrinsic ones like island size, distance to the mainland and the amount of available habitat. Another group of factors comprise intrinsic properties such as mutation rate, dispersal capacity, diversity of the population of provenance, and the capacity to respond to human disturbance (Stuessy, Takayama, Lopez‐Sepulveda, & Crawford, 2014). When all of these factors are taken into account, the low genetic diversity detected for *B. edulis* is not surprising. For example, although the total area of Guadalupe Island is about 240.00 km^2^, *B. edulis* occupies only 0.8 km^2^ at the foggy northern extreme of the Island (Garcillán et al., 2012; Oberbauer, 2005). This restricted available habitat reduces the potential establishment of new populations and limits effective population size (Frankham, 1996). Another important factor that may explain the low diversity of *B. edulis* is the low diversity of *Brahea* populations from the Baja Peninsula. For example, only two chloroplast haplotypes were detected in over 60 peninsular *Brahea* individuals. Additionally, the long generation time of *Brahea* (Bullock & Heath, 2006) and low mutation rates in the Arecaceae (Wilson et al., 1990) may both limit the rate at which new genetic variation is acquired by *B. edulis* (Stuessy et al., 2014).

### 4.3 Congruence with historical scenarios

During the last six million years, the Baja California Peninsula has undergone many geological changes that have profoundly affected the distribution and genetic structure of plant and animals species (Dolby et al., 2015; Riddle et al., 2000). Most notably, the formation of the Gulf of California and separation of the Baja California Peninsula from the Mexican mainland, which began approximately 6 million years ago, resulted in the complete or partial isolation of many plant and animal populations on either side of the break (Dolby et al., 2015; Garrick et al., 2009; Gutiérrez Flores et al., 2016; Riddle et al., 2000). Currently, the Gulf of California represents a major barrier to gene flow even for certain organisms with moderate dispersal capabilities (Grismer, 2000; Riddle et al., 2000), so in some respects it is unsurprising that it also appears to impeded gene flow in sessile organisms like plants (Cavender‐Bares et al., 2015). Consistent with this, we found a strong pattern of haplotype differentiation between mainland and peninsular *W. robusta* at both nuclear and chloroplast sequences. Our results from the chloroplast data for *Brahea* were similar, but as only one individual from the Mexican mainland was analyzed, we could not draw any firm conclusions. Nonetheless, although we lacked samples from the Mexican mainland for *Brahea*, we were able to include an endemic *Brahea* palm species from Guadalupe Island. This volcanic island has never been in contact with the Baja Peninsula and as a consequence almost 16% of its native plant species are endemics (Batiza, 1977; León De La Luz et al., 2003). Deep nuclear isolation of Guadalupe Island's *Brahea* from its peninsular sister species is consistent with a strong effect of dispersal barriers like sea channels on the population structure of *Brahea* palms (Karhu et al., 2005; Rosas‐Escobar et al., 2011).

Historically, the Baja has also undergone a series of submersions and uplifts (Lindell et al., 2006; Riddle et al., 2000). During these partial submersions, the Peninsula was fragmented into one or more islands and the Pacific Ocean was connected to the Gulf of California. More specifically, inundation of the Isthmus of La Paz isolated the Cabo region (SL) from the rest of the Baja Peninsula, whereas the mid‐peninsular seaway temporally isolated the southern and northern parts of the Baja Peninsula (Lindell et al., 2006; Riddle et al., 2000). Nonetheless, the exact locations of the proposed seaways as well as the timing of their formation are still under debate (Dolby et al., 2015). Regardless of the precise time and location estimates, these breaks are thought to be responsible for the observed patterns of genetic structuring in a variety of taxa (Garrick et al., 2009; Lindell, Méndez de la Cruz, & Murphy, 2005; Lira‐Noriega, Toro‐Núñez, Oaks, & Mort, 2015; Riddle et al., 2000; Upton & Murphy, 1997), although alternative explanations have also been proposed (Gutiérrez Flores et al., 2016). *Washingtonia's* nuclear and plastid sequence data did not provide any support for either of these breaks, with clustering results indicating the presence of a single genetically homogeneous population along the peninsula. By contrast, for Brahea, the chloroplast data strongly supported the presence of two genetically differentiated clades along the peninsula (Figures 3a,c, and 5b), providing support for historical marine intrusion into lowland areas of the Isthmus of La Paz.

## CONFLICT OF INTEREST

None declared.

## References

[ece33036-bib-0001] Aleixandre, P. , Hernandez Montoya, J. , & Mila, B. (2013). Speciation on oceanic islands: Rapid adaptive divergence vs. cryptic speciation in a Guadalupe Island songbird (Aves: *Junco*). PLoS One, 8(5), e63242. 23675466 10.1371/journal.pone.0063242 PMC3651090

[ece33036-bib-0002] Araújo, M. B. , & Luoto, M. (2007). The importance of biotic interactions for modelling species distributions under climate change. Global Ecology and Biogeography, 16, 743–753.

[ece33036-bib-0004] Axelrod, D. I. (1950). Classification of the Madro Tertiary Flora. Carnegie Institution of Washington Publication, 59, 1–22.

[ece33036-bib-0005] Bacon, C. D. , Baker, W. J. , & Simmons, M. P. (2011). Miocene dispersal drives island radiations in the palm tribe *Trachycarpeae* (Arecaceae). Systematic Biology, 61, 426–442. 10.1093/sysbio/syr123 22223444

[ece33036-bib-0006] Bacon, C. D. , Feltus, F. A. , Paterson, A. H. , & Bailey, C. D. (2008). Novel nuclear intron‐spanning primers for Arecaceae evolutionary biology. Molecular Ecology Resources, 8, 211–214. 21585759 10.1111/j.1471-8286.2007.01928.x

[ece33036-bib-0007] Baker, W. J. , & Couvreur, T. L. P. (2013). Global biogeography and diversification of palms sheds light on the evolution of tropical lineages. Historical biogeography. Journal of Biogeography, 40, 274–285.

[ece33036-bib-0008] Barrett, C. F. , Bacon, C. D. , Antonelli, A. , Cano, A. , & Hofmann, T. (2016). An introduction to plant phylogenomics, with a focus on palms. Botanical Journal of the Linnean Society, 182, 234–255. https://doi.org/10.1111/boj.12399

[ece33036-bib-0009] Batiza, R. (1977). Petrology and chemistry of Guadalupe Island: An alkalic seamount on a fossil ridge crest. Geology, 5, 760–764.

[ece33036-bib-0010] Bullock, S. H. , & Heath, D. (2006). Growth rates and age of native palms in the Baja California desert. Journal of Arid Environments, 67, 391–402.

[ece33036-bib-0011] Cavender‐Bares, J. , Gonzalez‐Rodriguez, A. , Eaton, D. A. R. , Hipp, A. A. L. , Beulke, A. , & Manos, P. S. (2015). Phylogeny and biogeography of the American live oaks (*Quercus* subsection *Virentes*): A genomic and population genetics approach. Molecular Ecology, 24(14), 3668–3687. 26095958 10.1111/mec.13269

[ece33036-bib-0012] CBOL Plant Working Group (2009). A DNA barcode for land plants. Proceedings of the National Academy of Sciences of the United States of America, 106, 12794–12797. 19666622 10.1073/pnas.0905845106 PMC2722355

[ece33036-bib-0013] Clark‐Tapia, R. , & Molina‐Freaner, F. (2003). The genetic structure of a columnar cactus with a disjunct distribution: *Stenocereus gummosus* in the Sonoran desert. Heredity, 90, 443–450. 12764419 10.1038/sj.hdy.6800252

[ece33036-bib-0014] Clement, M. , Posada, D. , & Crandall, K. A. (2000). TCS: Estimating gene genealogies. Molecular Ecology, 10, 1657–1666. 10.1046/j.1365-294x.2000.01020.x 11050560

[ece33036-bib-0015] Corander, J. , Marttinen, P. , Siren, J. , & Tang, J. (2008). Enhanced Bayesian modelling in BAPS software for learning genetic structures of populations. BMC Bioinformatics, 9, 539. 19087322 10.1186/1471-2105-9-539 PMC2629778

[ece33036-bib-0016] Corander, J. , Siren, J. , & Arjas, E. (2008). Bayesian spatial modelling of genetic population structure. Computational Statistics, 23, 111–129.

[ece33036-bib-0017] Corander, J. , Waldmann, P. , & Sillanpaa, M. J. (2003). Bayesian analysis of genetic differentiation between populations. Genetics, 163, 367–374. 12586722 10.1093/genetics/163.1.367 PMC1462429

[ece33036-bib-0018] Cornett, J. W. (1985). Reading the fan palms. Natural History, 94, 64–73.

[ece33036-bib-0020] Cornett, J. W. (1987a). Three palm species at Catavina. Principes (USA), 31(1), 12–13.

[ece33036-bib-0021] Cornett, J. W. (1987b). Status of desert fan palm populations in the Sonoran and Mojave deserts, In Abstracts: Symposium on the scientific value of the desert, Educational Bulletin 87‐1Sp. Spring Valley, Desert Protective Council:10.

[ece33036-bib-0022] Cornett, J. W. (2008). The desert fan palm oasis In Stevens L., & Meretsky V. J. (Eds.), Aridland springs in North America: Ecology and conservation (pp. 158–184). Tucson: The University of Arizona Press and the Arizona Sonora Desert Museum.

[ece33036-bib-0023] Cornett, J. W. , Glen, T. , & Stewart, J. M. (1986). The largest desert fan palm oases. Principes (USA), 30, 82–84.

[ece33036-bib-0024] Couvreur, T. L. P. , Forest, F. , & Baker, W. J. (2011). Origin and global diversification patterns of tropical rain forests: Inferences from a complete genus‐level phylogeny of palms. BMC Biology, 9, 44. 21679405 10.1186/1741-7007-9-44 PMC3142250

[ece33036-bib-0025] Currie, D. J. , Mittelbach, G. G. , Cornell, H. V. , Field, R. , Guegan, J. F. , Hawkins, B. A. , … Turner, J. R. G. (2004). Predictions and tests of climate‐based hypotheses of broad‐scale variation in taxonomic richness. Ecology Letters, 7, 1121–1134.

[ece33036-bib-0026] Dolby, G. A. , Bennett, S. E. K. , Lira‐Noriega, A. , Wilder, B. T. , & Munguia‐Vega, A. (2015). Assessing the geological and climatic forcing of biodiversity and evolution surrounding the Gulf of California. Journal of the Southwest, 57, 391–455.

[ece33036-bib-0027] Eiserhardt, W. L. , Svenning, J. C. , Kissling, W. D. , & Balslev, H. (2011). Geographical ecology of the palms (Arecaceae): Determinants of diversity and distributions across spatial scales. Annals of Botany, 108(8), 1391–1416. 21712297 10.1093/aob/mcr146 PMC3219491

[ece33036-bib-0028] Elith, J. , Phillips, S. J. , Hastie, T. , Dudík, M. , Chee, Y. E. , & Yates, C. J. (2011). A statistical explanation of maxent for ecologists. Diversity and Distributions, 17, 43–57.

[ece33036-bib-0030] Excoffier, L. , & Lischer, H. E. L. (2010). Arlequin suite ver 3.5: A new series of programs to perform population genetics analyses under Linux and Windows. Molecular Ecology Resources, 10, 564–567. 21565059 10.1111/j.1755-0998.2010.02847.x

[ece33036-bib-0031] Excoffier, L. , Smouse, P. E. , & Quattro, J. M. (1992). Analysis of molecular variance inferred from metric distances among mtDNA haplotypes: Application to human mitochondrial DNA restriction data. Genetics, 131, 479–491. 1644282 10.1093/genetics/131.2.479 PMC1205020

[ece33036-bib-0032] Felger R. S., & Broyles Y. B. (Eds.) (2007). Dry Borders: Great natural reserves of the Sonoran desert. Salt Lake City (p. 816). Utah: University of Utah Press.

[ece33036-bib-0033] Felger, R. S. , Johnson, M. B. , & Wilson, Y. M. F. (2001). The trees of Sonora, Mexico (p. 400). New York: Oxford University Press.

[ece33036-bib-0034] Felger, R. S. , & Joyal, E. (1999). The palms (Arecaceae) of Sonora, Mexico. Aliso: A Journal of Systematic and Evolutionary Botany, 18, 1–18.

[ece33036-bib-0035] Franco‐Vizcaino, E. , Lopez‐Beltran, A. C. , & Salazar‐Cesena, M. (2007). Water relations and community composition in three blue fan palm oases across the Californian‐Sonoran biome transition. The Southwestern Naturalist, 52, 191–200.

[ece33036-bib-0036] Frankham, R. (1996). Relationship of genetic variation to population size in wildlife. Conservation Biology, 10, 1500–1508.

[ece33036-bib-0037] Garcillán, P. P. , Vega, E. , & Martorell, C. (2012). The Brahea edulis palm forest in Guadalupe Island: A North American fog oasis? Revista Chilena de Historia Natural, 85, 137–145.

[ece33036-bib-0038] Garrick, R. C. , Nason, J. D. , Meadows, C. A. , & Dyer, R. J. (2009). Not just vicariance: Phylogeography of a Sonoran Desert euphorb indicates a major role of range expansion along the Baja Peninsula. Molecular Ecology, 18, 1916–1931. 19302467 10.1111/j.1365-294X.2009.04148.x

[ece33036-bib-0039] Grismer, L. L. (2000). Evolutionary biogeography on Mexico's Baja California peninsula: A synthesis of molecules and historical geology. Proceedings of the National Academy of Sciences of the United States of America, 97, 14017–14018. 11114205 10.1073/pnas.260509697 PMC34085

[ece33036-bib-0040] Grismer, L. L. , & McGuire, J. A. (1993). The oases of Central Baja California, Mexico. Part I. A preliminary account of the relict mesophytic herpetofauna and the status of the oases. Bulletin of the Southern California Academy of Sciences, 92, 2–24.

[ece33036-bib-0041] Gutiérrez Flores, C. , García‐De León, F. J. , León de la Luz, J. L. , & Cota‐Sánchez, J. H. (2016). Microsatellite genetic diversity and mating systems in the columnar cactus *Pachycereus pringlei* (Cactaceae). Perspectives in Plant Ecology, Evolution and Systematics., 22, 1–10.

[ece33036-bib-0042] Hafner, D. J. , & Riddle, B. R. (1997). Biogeography of Baja California peninsular desert mammals In Yates T. L., Gannon W. L. & Wilson D. E. (Eds.), Life among the muses: Papers in honor of James S. Findley (pp. 39–68). Albuquerque: The Museum of Southwestern Biology, University of New Mexico.

[ece33036-bib-0043] Hall, T. A. (1999). BioEdit: A user‐friendly biological sequence alignment editor and analysis program for Windows 95/98/NT. Nucleic Acids Symposium Series, 41, 95–98.

[ece33036-bib-0044] Hamilton, M. B. , & Miller, J. R. (2002). Relative rates of pollen and seed gene flow in the island model using nuclear and organelle measures. Genetics, 162, 1897–1909. 12524358 10.1093/genetics/162.4.1897 PMC1462371

[ece33036-bib-0045] Hamrick, J. L. , & Nason, J. D. (2000). Gene flow in forest trees In Boyle T. J. B., Young A., & Boshier D. (Eds.), Forest conservation genetics: Principles and practice (pp. 81–90). Collingwood, VIC, Australia: CIFOR and CSIRO.

[ece33036-bib-0046] Harley, M. M. (2006). A summary of fossil records for Arecaceae. Botanical Journal of the Linnean Society, 151, 39–67.

[ece33036-bib-0047] Henderson, A. , Galeano, G. , & Bernal, R. (1995). Field guide to the palms of the Americas (p. 498). Princeton, NJ: Princeton University Press.

[ece33036-bib-0048] Hewitt, G. M. (1996). Some genetic consequences of ice ages, and their role in divergence and speciation. Biological Journal of the Linnean Society, 58, 247–276.

[ece33036-bib-0049] Hewitt, G. M. (2000). The genetic legacy of the Quaternary ice ages. Nature, 405, 907–913. 10879524 10.1038/35016000

[ece33036-bib-0050] Hewitt, G. M. (2004). Genetic consequences of climatic oscillations in the Quaternary. Philosophical Transactions of the Royal Society of London B: Biological Sciences, 359, 183–195. 15101575 10.1098/rstb.2003.1388 PMC1693318

[ece33036-bib-0051] Hicks, B. F. (1989). Prehistoric development and dispersal of the desert fan palm. Principes (USA), 33, 33–39.

[ece33036-bib-0052] Hijmans, R. J. , Cameron, S. E. , Parra, J. L. , Jones, P. G. , & Jarvis, A. (2005). Very high resolution interpolated climate surfaces for global land areas. International Journal of Climatology, 25, 1965–1978.

[ece33036-bib-0053] Hodel, D. R. (2014). *Washingtonia*× filibusta (Arecaceae: Coryphoideae), a new hybrid from cultivation. Phytoneuron, 68, 1–7.

[ece33036-bib-0054] Jeanson, M. L. , Labat, J. N. , & Little, D. P. (2011). DNA barcoding: A new tool for palm taxonomists? Annals of Botany, 108, 1445–1451. 21757475 10.1093/aob/mcr158 PMC3219495

[ece33036-bib-0055] Karhu, A. , Vogl, C. , Moran, G. F. , Bell, J. C. , & Savolainen, O. (2005). Analysis of microsatellite variation in *Pinus radiata* reveals effects of genetic drift but no recent bottlenecks. Journal of Evolutionary Biology, 19, 167–175. 10.1111/j.1420-9101.2005.00982.x 16405588

[ece33036-bib-0056] Kerr, J. T. , & Packer, L. (1997). Habitat heterogeneity as a determinant of mammal species richness in high‐energy regions. Nature, 385, 252–254.

[ece33036-bib-0057] Kimura, M. (1980). A simple method for estimating evolutionary rate of base substitution through comparative studies of nucleotide sequences. Journal of Molecular Evolution, 16, 111–120. 7463489 10.1007/BF01731581

[ece33036-bib-0058] Kissling, W. D. , Baker, W. J. , Balslev, H. , Barfod, A. S. , Borchsenius, F. , Dransfield, J. , … Svenning, J. C. (2012). Quaternary and pre‐Quaternary historical legacies in the global distribution of a major tropical plant lineage. Global Ecology and Biogeography, 21(9), 909–921.

[ece33036-bib-0059] Kissling, W. D. , Eiserhardt, W. L. , Baker, W. J. , Borchsenius, F. , Couvreur, T. L. P. , Balslev, H. , & Svenning, J. C. (2012). Cenozoic imprints on the phylogenetic structure of palm species assemblages worldwide. Proceedings of the National Academy of Science of the United States of America, 109, 7379–7384. 10.1073/pnas.1120467109 PMC3358898 22529387

[ece33036-bib-0060] Kissling, W. D. , Rahbek, C. , & Bohning‐Gaese, K. (2007). Food plant diversity as broad‐scale determinant of avian frugivore richness. Proceedings of the Royal Society of London B: Biological Sciences, 274, 799–808. 10.1098/rspb.2006.0311 PMC2093978 17251107

[ece33036-bib-0061] Kondo, T. , Crisp, M. D. , Linde, C. , Bowman, D. M. J. S. , Kawamura, K. , Kaneko, S. , & Isagi, Y. (2012). Not an ancient relic: The endemic *Livistona* palms of arid central Australia could have been introduced by humans. Proceedings of the Royal Society of London B: Biological Sciences, 279, 2652–2661. 10.1098/rspb.2012.0103 PMC3350701 22398168

[ece33036-bib-0062] León De La Luz, J. L. , Rebman, J. P. , & Oberbauer, T. (2003). On the urgency of conservation on Guadalupe Island, Mexico: Is it a lost paradise? Biodiversity and Conservation, 12, 1073–1082.

[ece33036-bib-0063] León‐De la Luz, J. L. , & Breceda, A. (2006). Using endemic plant species to establish critical habitats in the Sierra de La Laguna Biosphere Reserve, Baja California Sur, Mexico. Biodiversity and Conservation, 15, 1043–1055.

[ece33036-bib-0064] Librado, P. , & Rozas, J. (2009). DnaSP v5: A software for comprehensive analysis of DNA polymorphism data. Bioinformatics, 25, 1451–1452. 19346325 10.1093/bioinformatics/btp187

[ece33036-bib-0065] Lindell, J. F. , Méndez de la Cruz, R. , & Murphy, R. W. (2005). Deep genealogical history without population differentiation: Discordance between mtDNA and allozyme divergence in the zebra‐tailed lizard (*Callisaurus draconoides*). Molecular Phylogenetics and Evolution, 36, 682–694. 15964216 10.1016/j.ympev.2005.04.031

[ece33036-bib-0066] Lindell, J. , Ngo, A. , & Murphy, R. W. (2006). Deep genealogies and the mid peninsular seaway of Baja California. Journal of Biogeography, 33, 1327–1331.

[ece33036-bib-0067] Lira‐Noriega, A. , Toro‐Núñez, O. , Oaks, J. R. , & Mort, M. E. (2015). The roles of history and ecology in chloroplast phylogeographic patterns of the bird‐dispersed plant parasite Phoradendron californicum (Viscaceae) in the Sonoran Desert. American Journal of Botany, 102(1), 149–164. 25587157 10.3732/ajb.1400277

[ece33036-bib-0068] Luikart, G. , England, P. R. , Tallmon, D. , Jordan, S. , & Taberlet, P. (2003). The power and promise of population genomics: From genotyping to genome typing. Nature Reviews Genetics, 4, 981–994. 10.1038/nrg1226 14631358

[ece33036-bib-0069] Mantooth, S. J. , Hafner, D. J. , Bryson, R. W. , & Riddle, B. R. (2013). Phylogeogrpahic diversification of antelope squirrels (*Ammospermophilus*) across North American deserts. Biological Journal of the Linnean Society, 109, 949–967.

[ece33036-bib-0070] McCauley, R. A. , Cortés‐Palomec, A. C. , & Oyama, K. (2010). Distribution, genetic structure, and conservation status of the rare microendemic species, *Guaiacum unijugum* (Zygophyllaceae) in the Cape Region of Baja California, Mexico. Revista Mexicana de Biodiversidad, 81(3), 745–758.

[ece33036-bib-0071] McClenaghan, L. , & Beauchamp, A. (1986). Low genic differentiation among isolated populations of the California fan palm (*Washingtonia filifera*). Evolution, 40, 315–322. 28556029 10.1111/j.1558-5646.1986.tb00473.x

[ece33036-bib-0072] McClintock, E. (1978). The Washington fan palm. Fremontia, 6, 3–5.

[ece33036-bib-0073] Minnich, R. A. , Franco‐Vizcaino, E. , & Salazar‐Ceseña, J. M. (2011). Distribution and regional ecology of Californian palm oases interpreted from Google earth images. Aliso: A Journal of Systematic and Evolutionary Botany, 29(1), 1–12.

[ece33036-bib-0074] Moran, R. (1996). The flora of Guadalupe Island, Mexico. Memoirs of the California Academy of Sciences, 19, 1–190.

[ece33036-bib-0075] Narum, S. R. , Buerkle, C. A. , Davey, J. W. , Miller, M. R. , & Hohenlohe, P. (2013). Genotyping‐by‐sequencing in ecological and conservation genomics. Molecular Ecology, 22(11), 2841–2847. 23711105 10.1111/mec.12350 PMC3935057

[ece33036-bib-0076] Nason, J. D. , Hamrick, J. L. , & Fleming, T. T. (2002). Historical vicariance and postglacial colonization effects on the evolution of genetic structure in *Lophocereus*, a Sonoran Desert columnar cactus. Evolution, 56, 2214–2226. 12487352 10.1111/j.0014-3820.2002.tb00146.x

[ece33036-bib-0077] Oberbauer, T. (2005). A comparison of estimated historic and current vegetation community structure on Guadalupe Island, Mexico In Garcelon D. K. & Schwemm C. A. (Eds.), Proceedings of the sixth California Islands Symposium (Pp. 143–153). Arcata, CA: National Park Service Technical Publication CHIS‐05‐01, Institute for Wildlife Studies.

[ece33036-bib-0078] Otto‐Bliesner, B. L. , Marshall, S. J. , Overpeck, J. T. , Miller, G. H. , Hu, A. , & CAPE Last Interglacial Project members . (2006). Simulating Arctic climate warmth and icefield retreat in the last interglaciation. Science, 311, 1751–1753. 16556838 10.1126/science.1120808

[ece33036-bib-0079] Parks, D. H. , Porter, M. , Churcher, S. , Wang, S. , Blouin, C. , Whalley, J. , … Beiko, R. (2009). Gengis: Ageospatial information system for genomic data. Genome Research, 19, 1896–1904. 19635847 10.1101/gr.095612.109 PMC2765287

[ece33036-bib-0080] Peterson, A. T. , & Nakazawa, Y. (2008). Environmental data sets matter in ecological niche modelling: An example with *Solenopsis invicta* and *Solenopsis richteri* . Global Ecology and Biogeography, 17, 135–144.

[ece33036-bib-0081] Petit, R. J. , Duminil, J. , Fineschi, S. , Hampe, A. , Salvini, D. , & Vendramin, G. G. (2005). Comparative organization of chloroplast, mitochondrial and nuclear diversity in plant populations. Molecular Ecology, 14, 689–701. 15723661 10.1111/j.1365-294X.2004.02410.x

[ece33036-bib-0082] Phillips, S. J. (2017). A brief tutorial on Maxent. Retrieved from http://biodiversityinformatics.amnh.org/open_source/maxent/ (accessed on 25 March 2017)

[ece33036-bib-0083] Phillips, S. J. , Anderson, R. P. , & Schapire, R. E. (2006). Maximum entropy modeling of species geographic distributions. Ecological Modelling, 190, 231–259.

[ece33036-bib-0084] Pillon, Y. , Johansen, J. B. , Sakishima, T. , Roalso, E. H. , Price, D. K. , & Stacy, E. A. (2013). Gene discordance in phylogenomics or recent plant radiations, an example from Hawaiian *Cyrtandra* (Gesneriaceae). Molecular Phylogenetics and Evolution, 69, 293–298. 23685062 10.1016/j.ympev.2013.05.003

[ece33036-bib-0085] Riddle, B. R. , Hafner, D. J. , Alexander, L. F. , & Jaeger, J. R. (2000). Cryptic vicariance in the historical assembly of a Baja California peninsular desert biota. Proceedings of the National Academy of Sciences of the United States of America, 97, 14438–14443. 11095731 10.1073/pnas.250413397 PMC18937

[ece33036-bib-0086] Riemann, H. , & Ezcurra, E. (2005). Plant endemism and natural protected areas in the peninsula of Baja California, Mexico. Biological Conservation, 122, 141–150.

[ece33036-bib-0087] Rosas‐Escobar, P. , Gernandt, D. S. , Pinero, D. , & Garcillan, P. P. (2011). Plastid DNA diversity is higher in the island endemic Guadalupe Cypress than in the continental Tecate Cypress. PLoS One, 6(1), e16133. 21283771 10.1371/journal.pone.0016133 PMC3024418

[ece33036-bib-0088] Schoener, T. W. (1968). Anolis lizards of Bimini: Resource partitioning in a complex fauna. Ecology, 49, 704–726.

[ece33036-bib-0089] Shaw, J. , Lickey, E. B. , Beck, J. T. , Farmer, S. B. , Liu, W. , Miller, J. , … Small, R. L. (2005). The tortoise and the hare II: Relative utility of 21 noncoding chloroplast DNA sequences for phylogenetic analysis. American Journal of Botany, 92, 142–166. 21652394 10.3732/ajb.92.1.142

[ece33036-bib-0090] Stuessy, T. F. , Takayama, K. , Lopez‐Sepulveda, P. , & Crawford, D. J. (2014). Interpretations of patterns of genetic variation within endemic plant species of oceanic islands. Botanical Journal of the Linnean Society, 174, 276–288. 26074627 10.1111/boj.12088 PMC4459035

[ece33036-bib-0091] Svenning, J. C. , Normand, S. , & Skov, F. (2008). Postglacial dispersal limitation of widespread forest plant species in nemoral Europe. Ecography, 31, 316–326.

[ece33036-bib-0092] Svenning, J. C. , & Skov, F. (2007). Ice age legacies in the geographical distribution of tree species richness in Europe. Global Ecology and Biogeography, 16, 234–245.

[ece33036-bib-0093] Swets, J. A. (1988). Measuring the accuracy of diagnostic systems. Science, 240, 1285–1293. 3287615 10.1126/science.3287615

[ece33036-bib-0094] Taberlet, P. , Gielly, L. , Pautou, G. , & Bouvet, J. (1991). Universal primers for amplification of three non‐coding regions of chloroplast DNA. Plant Molecular Biology, 17, 1105–1109. 1932684 10.1007/BF00037152

[ece33036-bib-0095] Templeton, A. R. , Crandall, K. A. , & Sing, C. F. (1992). A cladistic analysis of phenotypic associations with haplotypes inferred from restriction endonuclease mapping and DNA sequence data. III. Cladogram estimation. Genetics, 132, 619–633. 1385266 10.1093/genetics/132.2.619 PMC1205162

[ece33036-bib-0096] Tomlinson, P. B. (2006). The uniqueness of palms. Botanical Journal of the Linnean Society, 151, 5–14.

[ece33036-bib-0097] Tregear, J. W. , Rival, A. , & Pintaud, J. C. (2011). A family portrait: Unravelling the complexities of palms. Annals of Botany, 108, 1387–1389. 22200064 10.1093/aob/mcr269 PMC3219500

[ece33036-bib-0098] Upton, D. E. , & Murphy, R. W. (1997). Phylogeny of the side‐blotched lizards (Phrynosomatidae: Uta) based on mtDNA sequences: Support for midpeninsular seaway in Baja California. Molecular Phylogenetics and Evolution, 8, 104–113. 9242598 10.1006/mpev.1996.0392

[ece33036-bib-0099] Warren, D. L. , Glor, R. E. , & Turelli, M. (2008). Environmental niche equivalency versus conservatism: Quantitative approaches to niche evolution. Evolution, 62, 2868–2883. 18752605 10.1111/j.1558-5646.2008.00482.x

[ece33036-bib-0100] Warren, D. L. , Glor, R. E. , & Turelli, M. (2010). ENMTools: A toolbox for comparative studies of environmental niche models. Ecography, 33, 607–611.

[ece33036-bib-0101] Watanabe, S. , Hajima, T. , Sudo, K. , Nagashima, T. , Takemura, T. , Okajima, H. , … Kawamiya, M. (2011). MIROC‐ESM: Model description and basic results of CMIP5‐20c3 m experiments. Geoscientific Model Development, 4, 1063–1128.

[ece33036-bib-0102] Wehncke, E. V. , & López‐Medellín, X. (2015). Living at the edge: Blue fan palm desert oases of northern Baja California In Ezcurra E., Lara R., Álvarez‐Borrego S. & Wehncke E. V. (Eds.), Conservation science of Mexico′s Northwest (pp. 311–330), México, D.F.: UCMexus, SEMARNAT y INECC‐Semarnat.

[ece33036-bib-0103] Wehncke, E. V. , López‐Medellín, X. , & Ezcurra, E. (2009). Patterns of Frugivory, Seed Dispersal and Predation of Blue Fan Palms (*Brahea armata*) in Oases of Northern Baja California. Journal of Arid Environments, 73(9), 773–783.

[ece33036-bib-0104] Wehncke, E. V. , López‐Medellín, X. , & Ezcurra, E. (2010). Blue fan palm distribution and seed removal patterns in three desert oases of northern Baja California, Mexico. Plant Ecology, 208(1), 1–20.

[ece33036-bib-0105] Wehncke, E. V. , López‐Medellín, X. , Wall, M. , & Ezcurra, E. (2013). Revealing an endemic herbivore‐palm interaction in remote desert oases of Baja California. American Journal of Plant Sciences, 4(2A), 470–478.

[ece33036-bib-0106] Weir, B. S. , & Cockerham, C. C. (1984). Estimating F‐statistics for the analysis of population struture. Evolution, 38, 1358–1370. 28563791 10.1111/j.1558-5646.1984.tb05657.x

[ece33036-bib-0107] Wiggins, I. L. (1980). Flora of Baja California (p. 1025). Stanford, CA: Stanford University Press.

[ece33036-bib-0108] Wilson, M. A. , Gaut, B. , & Clegg, M. T. (1990). Chloroplast DNA evolves slowly in the palm family (Arecaceae). Molecular Biology and Evolution, 7(4), 303–314. 1974691 10.1093/oxfordjournals.molbev.a040605

