# Supplementary material for: Molecular genetic analysis of two native desert palm genera, Washingtonia and Brahea, from the Baja California Peninsula and Guadalupe Island
Source: Ecol Evol. 2017 May 30;7(13):4919–35. doi: 10.1002/ece3.3036 (PMC5496553; doi:10.1002/ece3.3036)
Supplement: Supplementary file 1 [file ECE3-7-4919-s001.docx]

*Supplemental information.*

Supplemental Table 1. Geo-references for each sampling site included in the present study for *Washingtonia* and *Brahea* palms.

| Species | Sierra/popul | Sampling site | Latitude | Longitude |
| --- | --- | --- | --- | --- |
|  |  |  |  |  |
| *B. edulis* | Guadalupe Island (GI) | Sur | 28.9326278 | -118.2837389 |
|  |  | Pista | 29.0056861 | -118.2495361 |
|  |  | Norte | 29.1612556 | -118.3213333 |
| *B. brandegeei* | Cabo Region (SL) | Sierra Laguna 1 | 23.2218722 | -109.8695028 |
|  |  | Sierra Laguna 2 | 23.228125 | -109.8889611 |
|  |  | Cienegita | 23.2381111 | -109.9518333 |
|  |  | San Antonio 1 | 23.65115 | -109.92985 |
|  |  | San Antonio 2 | 23.6855528 | -109.9450639 |
|  |  | Sierra Cacachillas | 24.0813917 | -110.1125667 |
|  |  | Las Cruces | 24.2391583 | -110.2031806 |
|  | Sierra Mechudo (SM) | Pilar | 24.47165 | -111.0027889 |
|  |  | Mechudo_1 | 24.7703194 | -110.7470833 |
|  |  | Soledad | 24.8121389 | -110.8141306 |
|  | Sierra San Pedro (SSP) | SSP | 26.88801 | -112.43786 |
|  | San Fransisco de la Sierra (SSF) | Los Pilares | 27.5942083 | -113.0333444 |
|  |  | Santa Gertrudis | 28.0495 | -113.084225 |
| *B. armata* | Sierra Libertad (SLI) | Corrales | 28.3109583 | -113.5291361 |
|  |  | Sepetales | 28.354546 | -113.489647 |
|  |  | San Borjas | 28.7401417 | -113.7531278 |
|  | SierraAsamblea (SA) | Asamblea | 29.2616472 | -114.0187889 |
|  | Cataviña (Cat) | Santa Ines | 29.743054 | -114.673552 |
|  |  | Catavina_2 | 29.749688 | -114.742954 |
|  | Sierra San Pedro Mártir (SSPM) | Pinole 4 | 30.2765917 | -115.2237278 |
|  |  | Pinole 3 | 30.3169639 | -115.2629694 |
|  |  | Pinole 2 | 30.3240333 | -115.2055861 |
|  |  | San Simon | 30.4983778 | -115.7677306 |
|  | Sierra Juárez (SJ) | Guadalupe Canyon | 32.1535667 | -115.7888833 |
|  |  | Tajo Canyon | 32.2634111 | -115.8383611 |
| *B. elegans* | Sonora | Arroyo Santana | 28.350000 | -109.25000 |
| *W. robusta* | Cabo Region (SL) | San Antonio1 | 23.65115 | -109.92985 |
|  |  | S. Bartolo | 23.7358694 | -109.8433778 |
|  |  | S. Vicente | 23.2783056 | -110.0375556 |
|  |  | S. Cacachillas | 24.0813917 | -110.1125667 |
|  |  | Las cruces | 24.2391583 | -110.2031806 |
|  | Sierra Mechudo  (SM) | Km96 | 24.3339861 | -110.9142389 |
|  |  | Mechudo_3 | 24.4493972 | -110.7975083 |
|  |  | Pilar | 24.47165 | -111.0027889 |
|  |  | Mechudo_2 | 24.4929972 | -110.8073778 |
|  |  | Animas | 24.4941611 | -110.8123583 |
|  |  | Mechudo_1 | 24.7703194 | -110.7470833 |
|  |  | Soledad | 24.8121389 | -110.8141306 |
|  |  | Cantarranas | 24.8485222 | -111.0791306 |
|  |  | Banderita | 24.9908611 | -110.964311 |
|  | Sierra La Giganta (SG) | San Fransisco L | 25.7176722 | -111.4340167 |
|  |  | El Peloteado | 25.755778 | -111.516958 |
|  |  | Primera Agua | 25.935722 | -111.409793 |
|  |  | Canyon Parras | 25.97617 | -111.466954 |
|  |  | Comondu | 26.0169417 | -111.409793 |
|  | Sierra San Pedro (SSP) | Vieja mision | 26.9219139 | -112.4075167 |
|  | Sierra Aguaje (Son) | Nacapule | 28.0170417 | -111.0553167 |
|  |  | Manga | 28.0069472 | -111.1305389 |
|  | San Fransisco de la Sierra (SSF) | Santa Gertrudis | 28.0495 | -113.084225 |
|  | Sierra Libertad (SLI) | San Borjas | 28.7401417 | -113.7531278 |
|  | Cataviña  (Cat) | Santa Ines | 29.743054 | -114.673552 |
|  |  | Catavina_2 | 29.749688 | -114.742954 |
| *W. filifera* | Sierra Juárez (SJ) | Canyon Guadalupe | 32.1535667 | -115.7888833 |
|  |  | Tajo Canyon | 32.2634111 | -115.8383611 |
|  |  | Cañada | 32.3515944 | -115.8281667 |

Supplemental Table 2. Estimates of genetic differentiation among three taxonomically (Minich et al. 2011) designated species of *Brahea* based on the nuclear data. Pairwise *F*_st_ and Φ_st_ values are shown above and below the diagonal respectively. FDR corrected *P*-values: * *P* < 0.05; ** *P* <0.01; *** *P* < 0.001.

| Species | *B. brandegeei* | *B.armata* | *B.edulis* |
| --- | --- | --- | --- |
| *B. brandegeei* | - | 0.26*** | 0.81*** |
| *B.armata* | 0.13*** | - | 0.83*** |
| *B.edulis* | 0.55*** | 0.57** | - |

Supplemental Table 3. Results of analyses of molecular variance (AMOVA) for *Washingtonia spp*. based on (a) chloroplast; and (b) nuclear data, with the dataset being partitioned into taxonomically designated species, three major regions and eight sierras.

| (a) Chloroplast | Partition | Source of Variation | % of total variance | *F* | *P* |
| --- | --- | --- | --- | --- | --- |
|  | Current taxonomy | Among species | 94.8 | 0.95 | <0.001 |
|  |  | Among sites within species | 2.3 | 0.44 | <0.001 |
|  |  | Within sites | 2.9 | 0.97 | <0.001 |
|  | 3 regions | Among regions | 94.8 | 0.95 | <0.001 |
|  |  | Among sites within regions | -0.2 | -0.04 | 0.68 |
|  |  | Within sites | 5.4 | 0.95 | <0.001 |
|  | 8 sierras | Among sierras | 88.6 | 0.89 | <0.001 |
|  |  | Within sierras | 11.4 | – | – |
| (b)  Nuclear | Partition | Source of Variation | % of total variance | *F* | *P* |
|  | Current taxonomy | Among species | 80.2 | 0.80 | 0.016 |
|  |  | Among sites within species | 9.8 | 0.49 | <0.001 |
|  |  | Within sites | 9.9 | 0.9 | <0.001 |
|  | 3 regions | Among regions | 87.1 | 0.87 | 0.007 |
|  |  | Among sites within regions | -0.6 | -0.05 | 0.98 |
|  |  | Within sites | 13.5 | 0.87 | <0.001 |
|  | 8 sierras | Among  sierras | 70.6 | 0.71 | <0.001 |
|  |  | Within sierras | 29.4 | - |  |

Supplemental Table 4. Results of analyses of molecular variance (AMOVA) for *Brahea spp*. based on the nuclear data, with the dataset being partitioned into taxonomically designated species, *B.brandeegei* being restricted to the Cabo region and ten sierras.

| *Brahea* | Partition | Source of Variation | % of total variance | *F* | *P* |
| --- | --- | --- | --- | --- | --- |
|  | Current taxonomy | Among species | 62.87 | 0.62 | <0.001 |
|  |  | Among sites within species | 9.02 | 0.24 | 0.001 |
|  |  | Within sites | 28.12 | 0.72 | <0.001 |
|  | *B.brandegeei* restricted to Cabo | Among species | 50.50 | 0.51 | 0.03 |
|  |  | Among sites within species | 21.22 | 0.43 | <0.001 |
|  |  | Within sites | 28.27 | 0.72 | <0.001 |
|  | 10  sierras | Among sierras | 65.9 | 0.66 | <0.001 |
|  |  | Within sierras | 34.1 | – | – |

Supplemental Table 5. The relative contributions of different environmental variables to the Maxent models of *Washingtonia* and *Brahea* contemporary distributions.

| Code | Environmental variables | *Washingtonia*, % contribution | *Brahea*, % contribution |
| --- | --- | --- | --- |
| Bio8 | Mean Temperature of Wettest Quarter | 16.2 | 3.9 |
| Bio9 | Mean Temperature of Driest Quarter | 3.2 | 11.1 |
| Bio10 | Mean Temperature of Warmest Quarter | 8.2 | 14.5 |
| Bio11 | Mean Temperature of Coldest Quarter | 17.1 | 10.2 |
| Bio16 | Precipitation of Wettest Quarter | 14.3 | 9.2 |
| Bio17 | Precipitation of Driest Quarter | 37.2 | 42.0 |
| Bio19 | Precipitation of Coldest Quarter | 3.9 | 9.2 |

Supplemental Figure 1. Haplotype accumulation curves with 95% confidence intervals displayed as error bars. Panel (a) shows a haplotype accumulation curve for plastid sequences and panel (b) for nuclear sequences.

Supplemental Figure 2. Bayesian clustering of *Washingtonia* and *Brahea* individuals as estimated with BAPS: (a) chloroplast and (b) nuclear data. The abbreviations: BB (Cabo) - B*. brandegeei* from Sierra Laguna, BEL - *B.elegans*, BB - *B.brandegeei*, BA - *B.armata*, BE - *B.edulis*, WF – *W.filifera*, WR - *W.robusta*.

Supplemental Figure 3. Niche identity test plots between *Washingtonia* and *Brahea* quantified using (a) the standardized Hellinger distance (I) and (b) Schoener’s D. The vertical lines in each plot represent the actual values of niche overlap, while the histograms represent those of 1,000 pseudoreplicates.
